# Climatic Factors Drive Population Divergence and Demography: Insights Based on the Phylogeography of a Riparian Plant Species Endemic to the Hengduan Mountains and Adjacent Regions

**DOI:** 10.1371/journal.pone.0145014

**Published:** 2015-12-21

**Authors:** Zhi-Wei Wang, Shao-Tian Chen, Ze-Long Nie, Jian-Wen Zhang, Zhuo Zhou, Tao Deng, Hang Sun

**Affiliations:** 1 Key Laboratory for Plant Diversity and Biogeography of East Asia, Kunming Institute of Botany, Chinese Academy of Sciences, Kunming, 650201, China; 2 University of Chinese Academy of Sciences, Beijing, 100049, China; Technical University in Zvolen, SLOVAKIA

## Abstract

Quaternary climatic factors have played a significant role in population divergence and demography. Here we investigated the phylogeography of *Osteomeles schwerinae*, a dominant riparian plant species of the hot/warm-dry river valleys of the Hengduan Mountains (HDM), Qinling Mountains (QLM) and Yunnan-Guizhou Plateau (YGP). Three chloroplast DNA (cpDNA) regions (*trn*D-*trn*T, *psb*D-*trn*T, *pet*L-*psb*E), one single copy nuclear gene (glyceraldehyde 3-phosphate dehydrogenase; *G3pdh*), and climatic data during the Last Interglacial (LIG; *c*. 120–140 ka), Last Glacial Maximum (LGM; *c*. 21 ka), and Current (*c*. 1950–2000) periods were used in this study. Six cpDNA haplotypes and 15 nuclear DNA (nDNA) haplotypes were identified in the 40 populations of *O*. *schwerinae*. Spatial Analysis of Molecular Variance, median-joining networks, and Bayesian phylogenetic trees based on the cpDNA and nDNA datasets, all suggested population divergence between the QLM and HDM-YGP regions. Our climatic analysis identified significant heterogeneity of the climatic factors in the QLM and HDM-YGP regions during the aforementioned three periods. The divergence times based on cpDNA and nDNA haplotypes were estimated to be 466.4–159.4 ka and 315.8–160.3 ka, respectively, which coincide with the time of the weakening of the Asian monsoons in these regions. In addition, unimodal pairwise mismatch distribution curves, expansion times, and Ecological Niche Modeling suggested a history of population expansion (rather than contraction) during the last glaciation. Interestingly, the expansion times were found being well consistent with the intensification of the Asian monsoons during this period. We inferred that the divergence between the two main lineages is probably caused by disruption of more continuous distribution because of weakening of monsoons/less precipitation, whilst subsequent intensification of the Asian monsoons during the last glaciation facilitated the expansion of *O*. *schwerinae* populations.

## Introduction

**Fig 1:**
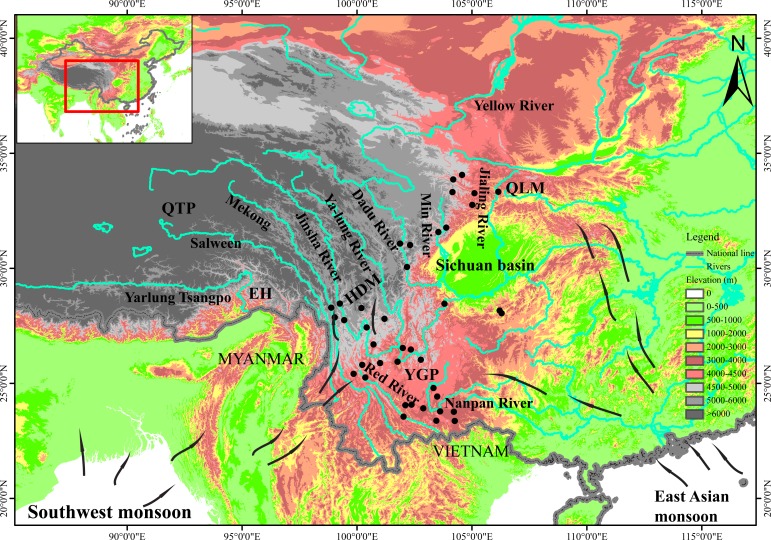
Topography, monsoons, and locations of sampled *Osteomeles schwerinae* populations in the involved regions.

Exploring factors associated with population processes (e.g. population divergence and demography) is an important issue in ecology and evolution [1–4]. Divergent selection and adaptation associated with different climatic factors have increasingly become recognized to have major influence on population demography, divergence and/or speciation in plants [4–7]. In recent years, a considerable number of phylogeographic studies have been conducted in the Hengduan Mountains (HDM) and adjacent regions [e.g. the Qinghai-Tibetan Plateau (QTP); Eastern Himalayas (EH); Yunnan-Guizhou Plateau (YGP); Qinling Mountains (QLM); Fig 1]. These studies provide robust molecular evidence that (sub)alpine plants presently occurring in these regions are strongly affected by glacial-interglacial climatic fluctuations during the Quaternary [8–14].

Hot/warm-dry river valleys are one of the most prominent natural landscape features in the HDM and adjacent regions [15]. In comparison to studies of (sub)alpine plants, however, few riparian plants of hot/warm-dry river valleys in these regions have been studied. The phylogeographic studies of riparian plants conducted so far have focused on two species: *Terminalia franchetii* (Combretaceae) and *Buddleja crispa* (Buddlejaceae), both of which indicated that the discontinuous distribution patterns of these plants are strongly linked to historical drainage re-organization events [16, 17]. More phylogeographic studies of riparian plants in the HDM and adjacent regions (particularly the QLM) are required to obtain a better understanding of their recent population history; and in particular, to detect whether some riparian species in these regions have been affected by climatic factors (i.e. heterogeneity and changes) during the Quaternary.

River valleys in different regions probably differ in climate because of different monsoon systems. The QLM, which is located on the north-eastern margin of the HDM (Fig 1), is dominated by the East Asian monsoon, originating from the Pacific Ocean [18, 19]. The east–west orientated mountains form a natural obstacle, preventing the wet summer East Asian monsoon from penetrating northern China, but provide good corridors for it to penetrate into the western QLM or even HDM. In contrast, The HDM is affected by both the southwest monsoon from the Indian Ocean and the East Asian monsoon from the Pacific Ocean, and the YGP is mainly affected by the East Asian monsoon [20]. Similarly, the south–north orientated river valleys in the HDM could also provide convenient corridors for the warm, moist air from the Indian and Pacific Oceans to penetrate into the northern and/or north-eastern HDM [21, 22]. During the Quaternary, the Asian monsoons appear to have fluctuated with the alternating interglacial-glacial periods, with more intense summer monsoons during interglacial periods and weaker monsoons during glaciations [23–25]. However, some strong summer monsoons seem to have also occurred during glacial periods (e.g. the last glaciation) in addition to their anticipated occurrence during interglacial periods. Conversely, some significantly weak summer monsoons have also been found to have occurred during interglacial periods (e.g. the last interglacial period) in addition to their occurrence during glacial periods [26–28]. Given the river valleys’ role as corridors for monsoons, it seems plausible that such climatic heterogeneity and changes due to Asian monsoons would have an impact on the population divergence and demography of riparian plants.

*Osteomeles schwerinae* Schneid. (Rosaceae) is an iconic riparian plant species endemic to the dry river valleys of the HDM, YGP, and QLM [29–31] (Fig 1), and probably ideal to study such impacts. It is a small tree species, characterized by imparipinnate leaves with 5 to 10 pairs of elliptic or elliptic-oblong leaflet blades, a campanulate hypanthium possessing sepals, and stony pomes [32]. According to the *Flora of China* [32] and our field observations, it commonly grows on the slopes of dry river valleys. It includes two varieties (*O*. *schwerinae* var. *schwerinae* and *O*. *schwerinae* var. *microphylla* Rehder & E. H. Wilson). The former is mainly confined to the river valleys of the HDM-YGP and has larger leaflet blades (5–10 mm), while the latter is mostly present in the river valleys of the QLM and has smaller leaflet blades (3–5 mm) [32]. Although there is now much molecular evidence, as mentioned above, that the population divergence and demographic history of taxa (e.g. alpine plants) in the HDM and adjacent regions were closely linked with historical climatic events [9, 11, 20], very little climatic data have been explicitly used to examine these inferences. In order to understand better how climatic factors contributed to the present-day distribution and population divergence of plants in the HDM and adjacent regions, we constructed the phylogeography of *O*. *schwerinae* based on molecular approaches and paleo-and current climatic data. We had the following objectives: (1) to quantify whether there is genetic divergence between the populations of *O*. *schwerinae*; (2) to explore whether the genetic divergence coincide with the regional morphological differentiation of *O*. *schwerinae* in the QLM and HDM-YGP regions; (3) to date divergences and determine whether these dates relate to geological events (e.g. historical drainage re-organization) or climatic changes (e.g. the monsoon fluctuation and/or the glacial-interglacial alternation); and (4) to unravel the demographic history of *O*. *schwerinae*.

## Material and Methods

### Ethics Statement

*Osteomeles schwerinae* is not protected nor endangered in the sampled areas, and all samples were collected by researchers following current Chinese regulations. None of the sampled locations are privately owned or protected by law.

### Sample Collection, DNA Extraction, and Sequencing

We sampled 304 individuals from 40 populations of *O*. *schwerinae* covering its geographical range; out of these, eight populations originated from the QLM region, and 32 populations from the HDM-YGP region (Table 1). Individuals were randomly sampled and were at least 100 m apart within each population. Fresh green leaves were collected from each individual and dried in silica gel for DNA extraction.

**Table 1:** Details of sampled populations of *Osteomeles schwerinae*, haplotypes (num. of individuals) detected per population, and the assignment of each population to the identified regions and drainage basins.

Code	Regions	Drainages	Latitude (°N)	Longitude (°E)	Altitude (m)	cpDNA haplotypes (num. of individuals)	nDNA haplotypes (num. of individuals)
1	QLM	Jialing River	33.87	104.18	1484	H1(7) H4(1)	Hap3(2) Hap4(3) Hap5(3) Hap11 (4) Hap13 (4)
2	QLM	Jialing River	34.07	104.57	1595	H1(8)	Hap2(1) Hap4(4) Hap11(6) Hap13(5)
3	QLM	Jialing River	33.27	105.11	1005	H1(6) H4(2)	Hap2(1) Hap3(2) Hap4(1) Hap5(3) Hap11(6) Hap13(3)
4	QLM	Jialing River	32.76	105.01	1668	H1(6) H4(2)	Hap2(2) Hap3(4) Hap5(4) Hap11(6)
5	QLM	Jialing River	33.33	106.15	663	H1(3) H4(5)	Hap3(4) Hap4(5) Hap13(6) Hap14(1)
6	QLM	Jialing River	33.32	104.15	1480	H1(8)	Hap1(1) Hap2(2) Hap3(6) Hap4(5) Hap5(2)
7	QLM	Min River	31.78	103.89	1578	H1(4) H6(3)	Hap2(2) Hap4(1) Hap5(2) Hap11(6) Hap13(5)
8	QLM	Min River	31.58	103.54	1476	H1(6) H6(2)	Hap2(2) Hap4(4) Hap5(1) Hap11(9)
9	HDM-YGP	Dadu River	31.08	101.87	2018	H1(1) H4(5) H5(2)	Hap2(6) Hap3(7) Hap5(3)
10	HDM-YGP	Dadu River	30.06	102.17	1410	H4(8)	Hap5(3) Hap7(2) Hap11(5) Hap12(6)
11	HDM-YGP	Dadu River	31.01	102.31	1860	H2(2) H4(6)	Hap5(16)
12	HDM-YGP	Ya-lung River	27.81	101.2	2268	H2(1) H4(6)	Hap9(10) Hap10(4)
13	HDM-YGP	Jinsha River	26.54	102.08	1091	H2(6) H4(1)	Hap3(6) Hap10(8)
14	HDM-YGP	Jinsha River	28.47	99.25	2125	H2(8)	Hap3(7) Hap6(9)
15	HDM-YGP	Jinsha River	27.75	99.44	1964	H2(5) H4(3)	Hap3(10) Hap6(6)
16	HDM-YGP	Jinsha River	28.28	100.19	2328	H2(8)	Hap3(16)
17	HDM-YGP	Jinsha River	27.43	100.42	2322	H2(6) H3(2)	Hap6(12) Hap7(4)
18	HDM-YGP	Jinsha River	26.7	100.72	2015	H2(8)	Hap3(8) Hap10(8)
19	HDM-YGP	Jinsha River	25.94	101.76	1335	H2(7) H4(1)	Hap10(16)
20	HDM-YGP	Jinsha River	25.89	101	1485	H2(8)	Hap3(12)
21	HDM-YGP	Jinsha River	26.48	102.34	1756	H2(6) H4(2)	Hap3(8) Hap10(8)
22	HDM-YGP	Jinsha River	26.03	102.78	1958	H2(5)	Hap3(10)
23	HDM-YGP	Jinsha River	28.46	103.8	926	H2(7)	Hap3(7) Hap10(7)
24	HDM-YGP	Jinsha River	28.17	106.21	923	H4(8)	Hap3(16)
25	HDM-YGP	Jinsha River	28.05	106.29	184	H4(8)	Hap3(16)
26	HDM-YGP	Red River	25.81	100.23	1977	H2(5) H4(2)	Hap3(10) Hap8(2) Hap9(2)
27	HDM-YGP	Red River	25.42	99.85	2165	H2(6) H4(2)	Hap3(11) Hap10(5)
28	HDM-YGP	Red River	25.26	100.36	1359	H2(8)	Hap3(7) Hap6(6) Hap10(3)
29	HDM-YGP	Red River	24.06	102.1	1194	H4(8)	Hap9(8) Hap10(8)
30	HDM-YGP	Red River	23.56	102.02	508	H2(8)	Hap3(16)
31	HDM-YGP	Red River	23.92	102.88	1399	H2(2) H4(5)	Hap10(14)
32	HDM-YGP	Red River	23.38	103.45	1356	H2(8)	Hap3(7) Hap10(9)
33	HDM-YGP	Nanpan River	24.8	103.33	1354	H2(7) H4(1)	Hap3(10) Hap10(6)
34	HDM-YGP	Nanpan River	24.43	103.49	1538	H2(6)	Hap10(12)
35	HDM-YGP	Nanpan River	24.08	102.37	1609	H4(7)	Hap3(2) Hap10(14)
36	HDM-YGP	Nanpan River	23.79	103.61	1467	H2(6)	Hap3(5) Hap10(7)
37	HDM-YGP	Nanpan River	23.37	104.25	1505	H2(7)	Hap3(6) Hap10(8)
38	HDM-YGP	Nanpan River	23.77	104.21	1565	H2(7)	Hap10(14)
39	HDM-YGP	Mekong	28.3	98.88	2351	H4(8)	Hap10(6) Hap15(10)
40	HDM-YGP	Mekong	27.87	99.03	1935	H4(8)	Hap10(13) Hap15(3)

Total genomic DNA was extracted from approximately 15 mg dried leaf using an Axygen Plant Mini Kit (www.axygen.com). Universal primers and methods described in previous studies [33, 34] were used to amplify three chloroplast DNA (cpDNA) intergenic spacers (*trn*D-*trn*T, *psb*D-*trn*T, *pet*L-*psb*E). Nuclear DNA (nDNA), namely the single copy nuclear gene (glyceraldehyde 3-phosphate dehydrogenase; *G3pdh*), was amplified following the PCR procedure in Olsen and Schaal (1999) [35], using one self-designed primer combination (forward: 5′-TTCAGCCACCCAGAAGAC-3′, reverse: 5′- CGACAACGGATAACAATAC -3′). All PCR products were purified and sequenced by the Sangon Corporation, Shanghai, China.

### Molecular Data Analysis

Haplotypes of the combined three cpDNA sequences were produced in DnaSP v5.0 [36]. Method of Clark (1990) was used to identify the haplotypes for *G3pdh* heterozygotes. In this method, sequences of heterozygotes were compared with those of homozygotes until we could account for the observed combination of double-banded sites. ArcMap v9.3 (ESRI, Inc.) was used to plot the distribution of haplotypes on a relief map coving the range. Original map in the figures was downloaded from http://www.diva-gis.org/Data. The elevation dataset in the figures was derived from NASA’s SRTM data (http://www2.jpl.nasa.gov/srtm).

Spatial Analysis of Molecular Variance (SAMOVA) was conducted to analyze the spatial genetic structure of *O*. *schwerinae* using SAMOVA v1.0 [37]. This program uses a simulated annealing approach to define *K* groups of populations that are geographically homogenous and maximally differentiated from each other. In this analysis, we assumed that *K* varied from 2 to 13, and computed a corresponding *F*_CT_ index, which measures the proportion of genetic differentiation among the *K* groups [38]. The configuration with the *F*_CT_ value that achieves a plateau first is usually considered the optimal grouping of populations. An analysis of molecular variance (AMOVA) was conducted within ARLEQUIN v3.5 [39] to partition variation within and among defined groups and populations.

Genealogical relationships among haplotypes were inferred from an unrooted statistical parsimony network within Network v4.6.1.2 [40] (available at http://www.fluxus-engineering.com) using a median-joining method. Indels (gaps) were treated separately as binary states, namely single events, following Caicedo and Schaal (2004) [41]. Mononucleotide repeats and duplicated indels of sequences in this study were removed as they introduced homoplasious reticulations into the network, due to their high levels of bidirectional mutation [42]. Observed and inferred haplotypes were nested following the rules in Posada and Crandall (2001) [43].

Phylogenetic relationships among haplotypes were reconstructed by Bayesian inference. This phylogenetic analysis was conducted in MrBayes v3.1.2 [44] using substitution models (GTR+G) explored by Modeltest v3.7 [45]. Four independent Markov chain Monte Carlo analyses with runs of 10 million generations were carried out, sampling at every 1000 generations. The first 20% of generations was discarded as burn-in, while the remaining data was used to construct a 50% majority rule consensus tree. The posterior probabilities found in this consensus tree were given to evaluate the robustness of the trees.

The divergence time of haplotypes was estimated via a molecular dating method, and estimated under a strict molecular clock in BEAST v1.7.5 [46], using the substitution rate range for angiosperm species (cpDNA: 1.01–3.0×10^−9^ substitutions per site per year (s/s/y) [47, 48]; nDNA: 1.58–3.15×10^−8^ s/s/y [48]). Results from BEAST were compiled and visualized in the program Tracer v1.5 [49].

Pairwise mismatch distribution was carried out using ARLEQUIN to infer the demographic history of *O*. *schwerinae*. In this analysis, an expected distribution was generated from 1000 parametric bootstrap replicates to test ‘demographic expansion’ and ‘spatial expansion’ model, respectively [2]. Generally, a unimodal mismatch distribution curve indicates a recent population expansion, whereas a multimodal distribution usually suggests that populations are at demographic equilibrium. The populations for which the hypothesis of expansion was not rejected, the value for the mode of the mismatch distribution (Tau) was assessed by 1000 parametric bootstrap replicates in ARLEQUIN, and was converted into estimates of time since expansion (t, in years BP) using t = Tau/2μkg [2]. In this formula, μ is the substitution rate, and was assumed to be based on the substitution rate range for cpDNA mentioned above; k is the average sequence length used for analysis (2039 bp in this study); and g is the generation time in years, and was assumed to be 6 years here based on personal observations of on the age of first reproduction of *O*. *schwerinae* in cultivation at Kunming Botanical Garden.

### Climatic Data Analysis

Climatic data [50] that summarized temperature and precipitation information (S1 Table), were downloaded from the WorldClim database (http://www.worldclim.org/), and were used to identify climatic factors potentially associated with the genetic structure and demographic history identified in this study. DIVA-GIS v7.5 [51] was used to extract the 19 climatic factors during the LIG, LGM, and Current periods (S2 Table). The mean values and standard errors of these factors for populations of the QLM and HDM-YGP regions were calculated with SPSS v18.0.0 [52], and were further analyzed using independent-Samples t-tests to validate the impact of the climatic factors on the genetic differentiation between the QLM and HDM-YGP regions.

Ecological Niche Modeling (ENM) can simulate past species distributions, and further elucidate demographic history through time [53]. Therefore, ENM was also performed in this study to validate potential present and past climate envelopes for this species. Assuming the species has not changed with respect to its climatic preference, we re-constructed the range of *O*. *schwerinae* during the LIG, LGM, and Current periods. Species occurrence data for *O*. *schwerinae* were obtained from collection records from the three main herbaria in China (IBSC, KUN and PE; S3 Table) and sampling records collected in this study (Table 1). ENM based on the 19 BioClim factors and species occurrence data was performed using MAXENT v3.3.3k [54] with the default parameters for number of iterations (500) and convergence threshold (10^−5^). We set up 10 replicate runs in each analysis in order to ensure more reliable results. Meanwhile, in order to test the performance of each model, 25% of the data in each run was randomly chosen by MAXENT and compared with the model output created with the remaining data. To reduce the effects of spatial autocorrelation, duplicate records from the same locality were also removed. The area under the receiver operating characteristic curves (AUC) was used to compare model performance. An AUC value of 0.5 indicates that the performance of the model is no better than random, while values closer to 1.0 indicate better model performance [54].

## Results

### Chloroplast and Nuclear DNA Sequencing Data

Variations in the length of two sequenced regions of cpDNA were detected (953–958 bp, *trn*T-*trn*D; 658–697 bp, *psb*D-*trn*T), while the *pet*L-*psb*E was uniformly 449 bp long in our samples. Removal of mononucleotide repeats and duplicated indels for the alignment of three cpDNA regions from the 304 samples resulted in an overall alignment length of 2039 bp, and produced six cpDNA haplotypes (designated H1-6; S4 Table). The aligned sequences of the *G3pdh* gene were 550 bp long, and produced 15 nDNA haplotypes (designated Hap1-15) based on 18 nucleotide substitutions (S5 Table). These sequences were deposited in the GenBank database (accession numbers KT724331–KT724335, KT750890–KT750920).

### Genetic Divergence and Demography

**Fig 2:**
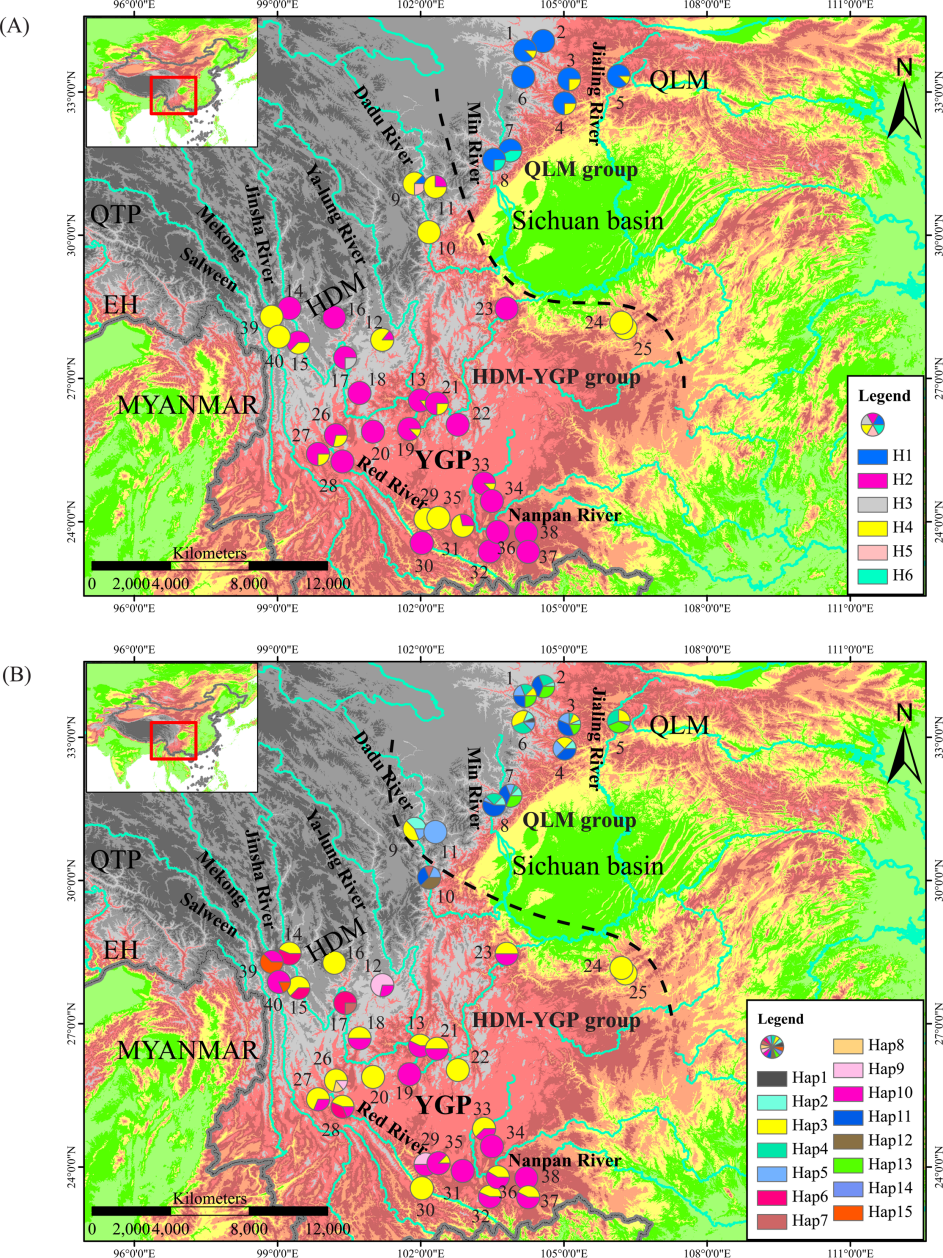
Geographical distributions of (A) six cpDNA haplotypes (H1-6) and (B) 15 nDNA haplotypes (Hap1-15) identified in 40 populations of *Osteomeles schwerinae*. Dashed black line delineates the boundary between the two groups derived from SAMOVA.

In the SAMOVA analysis based on cpDNA sequences, two groups corresponding to the QLM and HDM-YGP regions were well defined when K = 2. Although the *F*_CT_ value was not the highest, the *F*_CT_ values reached a plateau, and fluctuated little with an increasing number of groups (S1 Fig). In addition, when divided into two groups, the AMOVA revealed high genetic variation partitioned between the groups (68.57%), and low variations among populations within groups (18.60%) as well as within populations. As shown in Fig 2A, 8 populations located in the QLM region were contained in one group, whilst the remaining 32 populations from the HDM-YGP were included in the other group. The SAMOVA analysis based on nDNA sequences showed relatively low *F*_CT_ values, and fluctuated little with the increase of *K* (S2 Fig). Nonetheless, when divided into two groups, the AMOVA revealed that still 30.55% of the total nDNA variation was due to differences among the two groups identified, comparing with 22.61% among populations and 46.84% within populations. In this scenarios of grouping, 8 populations from the QLM (pops. 1–8) and 2 populations from the HDM-YGP (pops. 9 and 11 of the Dadu River) formed one group, whereas the remaining 30 populations from the HDM-YGP were included in the other group (Fig 2B).

**Fig 3:**
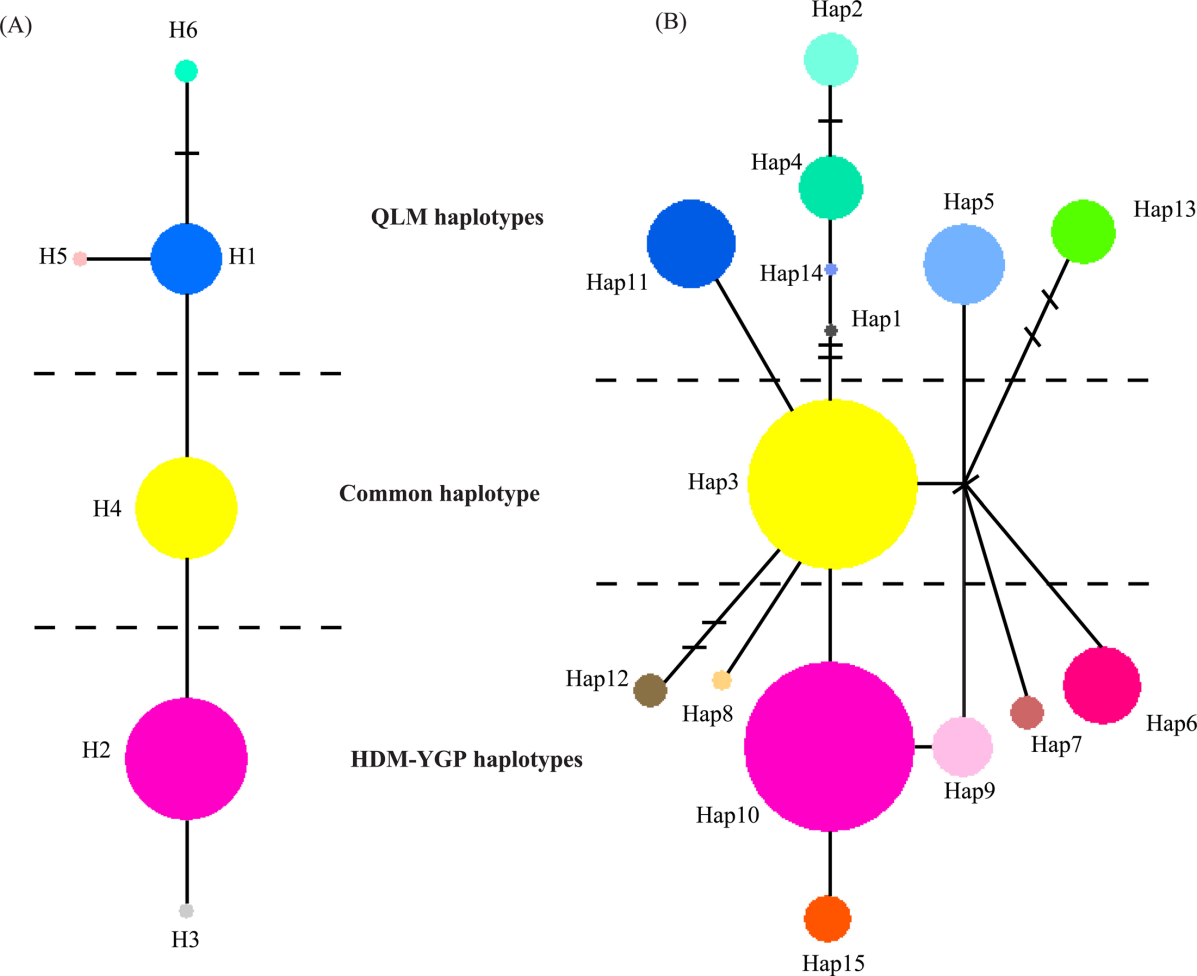
Median-joining networks of (A) six cpDNA haplotypes (H1-6) and (B) 15 nDNA haplotypes (Hap1-15) identified in this study. The size of circles corresponds to the frequency of each haplotype. Short lines represent haplotypes missing in the dataset and each branch represents one mutation.

Genealogical analysis of cpDNA haplotypes identified a common haplotype (H4) shared by populations along all rivers surveyed in QLM, HDM, and YGP regions (Fig 3A). Apart from this common haplotype, the remaining haplotypes were distinctly regional, and could be divided into two clusters from the QLM and HDM-YGP regions (Fig 3A). In addition, it is worth noting that the intermediate zone between these two regions (namely the drainage basin of the Dadu River) possess high cpDNA haplotype diversity, containing the common haplotype (H4), the main regional haplotypes from the QLM (H1) and HDM-YGP (H2), as well as its own unique haplotype (H5) (Table 1; Fig 2A). Genealogical analysis of nDNA haplotypes showed similar structure, which also identified a common haplotype (Hap3) and two clusters of regional haplotypes from the QLM and HDM-YGP, respectively (Fig 3B). Simultaneously, the intermediate zone between QLM and HDM-YGP (the Dadu River basin) also showed high nDNA haplotype diversity, possessing the common haplotype (Hap3), regional haplotypes both from the QLM (Hap2, 5, 11) and HDM-YGP (Hap7), as well as its own unique haplotype (Hap12; Table 1; Fig 2B).

**Fig 4:**
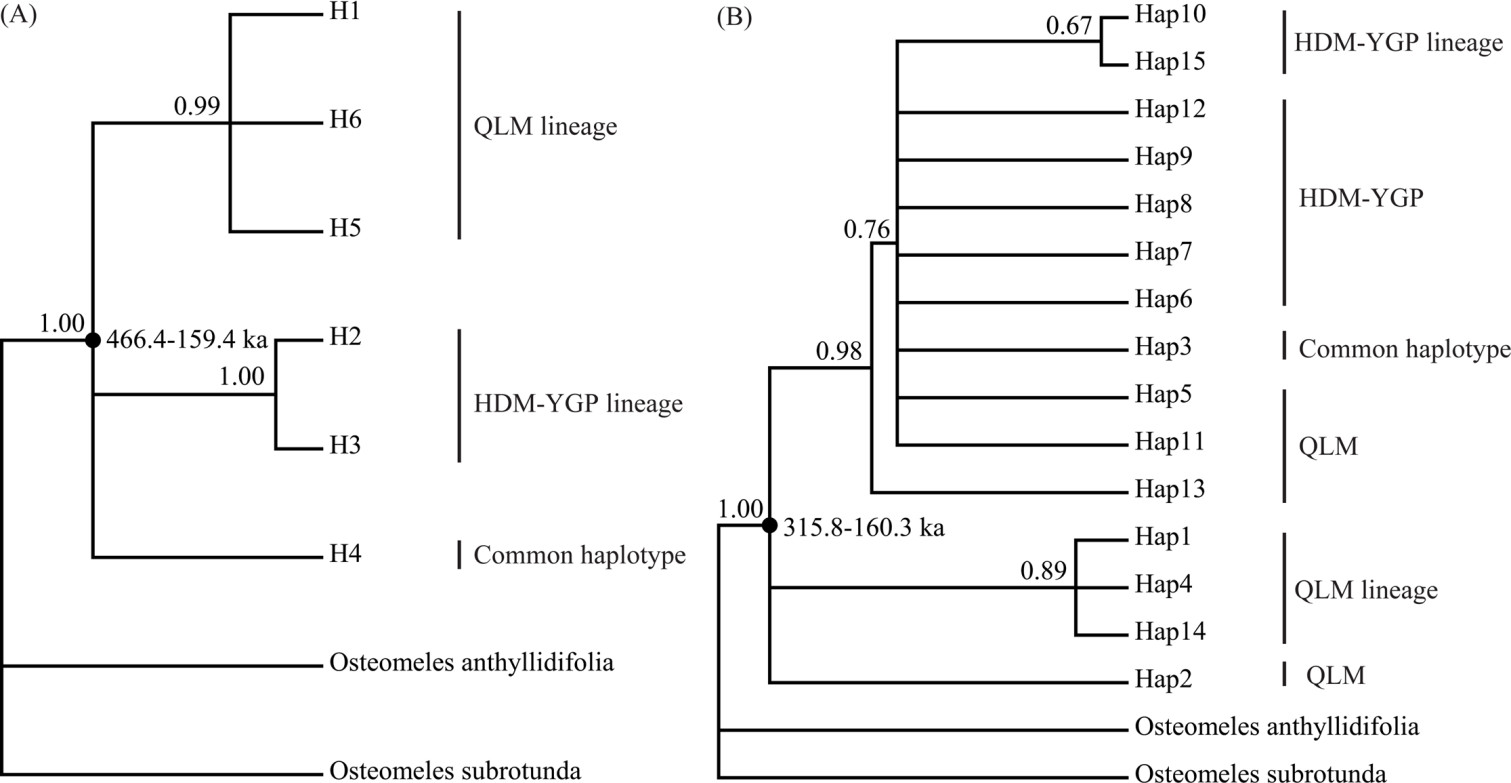
Bayesian phylogenetic trees of (A) six cpDNA haplotypes (H1-6) and (B) 15 nDNA haplotypes (Hap1-15) identified in this study. Numbers above the branch indicate posterior probabilities. Numbers next to the nodes indicate the divergence times.

The Bayesian phylogenetic tree clustered five of the cpDNA haplotypes (all except H4) into two lineages, respectively from the QLM and HDM-YGP regions (Fig 4A). In the phylogenetic tree based on nDNA haplotypes, one haplotype (Hap15) and the dominated haplotype (Hap10) in HDM-YGP region fell in one lineage, while three haplotypes (Hap 1, 4, 14) from QLM in another lineage (Fig 4B). In addition, this tree showed several polytomies, however, it is not surprising since the nuclear gene is two parents–inherited. Based on molecular dating, the divergence times of the cpDNA and nDNA haplotypes were estimated to be 466.4–159.4 ka and 315.8–160.3 ka, respectively.

**Fig 5:**
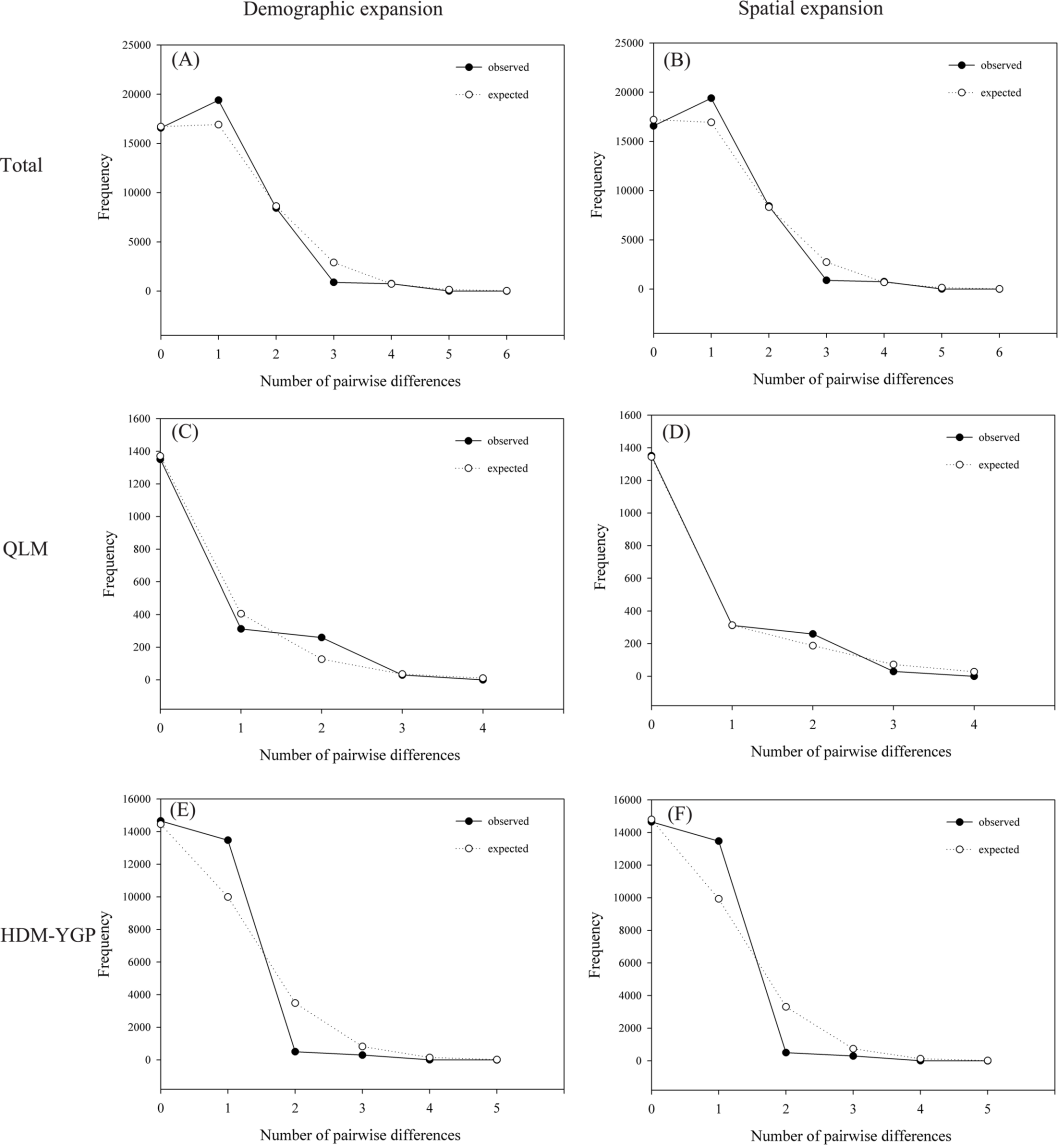
Mismatch distribution curves of the total (A, B) populations, QLM (C, D) populations, and HDM-YGP (E, F) populations. Left: demographic expansion; right: spatial expansion.

The mismatch distribution curves for the total, QLM and HDM-YGP populations were clearly unimodal (Fig 5). Therefore, the hypothesis of population expansion could not be rejected. Based on the Tau value, the demographic and spatial expansion times for the total, QLM and HDM-YGP populations fell within the last glacial period (Table 2).

**Table 2:** Results of the expansion time for the *Osteomeles schwerinae* populations.

Populations	Expansion types	Tau	Time since expansion began (ka)
Total	Demographic expansion	0.98438	13.41–39.83
	Spatial expansion	0.98510	13.42–39.86
QLM region	Demographic expansion	3.00000	40.87–121.39
	Spatial expansion	1.20902	16.47–48.93
HDM-YGP region	Demographic expansion	0.66992	9.13–27.11
	Spatial expansion	0.65968	8.98–26.69

### Climatic Data

Out of the 19 bioclimatic factors, all but five (bio2, 5, 8, 10, 15) during the LGM and the current periods showed highly significant differences in mean values between populations of the QLM and HDM-YGP regions (Table 3). When comparing with the LGM and Current periods, two more bioclimatic factors (bio8 and 15) during the LIG exhibited highly significant differences in mean values between the two regions (Table 3). As shown in Table 4, there was reduction in temperature during the LGM. For instance, the annual average temperature (bio1) in the QLM and HDM-YGP regions decreased 2.3 and 2.6°C during the LGM than the LIG, respectively. In contrast, the majority of the bioclimatic factors relating to the precipitation (6/8 = 75%) for both the QLM and HDM-YGP during the LGM period were higher than during the LIG, with most variables (5/6 = 83.33%) being significantly higher (Table 4). It suggested a more moist environment in the QLM and HDM-YGP regions during the LGM, whereas a more drought environment in the LIG.

**Table 3:** Means, standard errors (SE) and results of independent-Sample t-test on the 19 BioClim factors of the LIG, LGM, and Current periods for the QLM and HDM-YGP regions surveyed in this study.

Independent-Sample t-test		bio1	bio2	bio3	bio4	bio5	bio6	bio7	bio8	bio9	bio10	bio11	bio12	bio13	bio14	bio15	bio16	bio17	bio18	bio19
		(°C)	(°C)	(°C)	(SD*100)	(°C)	(°C)	(°C)	(°C)	(°C)	(°C)	(°C)	(mm)	(mm)	(mm)	(mm)	(mm)	(mm)	(mm)	(mm)
**LIG**																				
QLM	Mean	10.4	10.5	2.7	985.1	29.8	-8.4	38.3	14.3	1.0	22.8	-2.8	289.4	162.3	0.0	198.9	231.0	0.0	40.3	0.0
	SE	1.3	0.5	0.2	36.9	1.7	1.3	0.8	1.0	0.9	1.7	0.9	46.7	12.9	0.0	13.4	26.7	0.0	23.0	0.0
HDM-YGP	Mean	14.3	11.3	3.6	652.5	29.2	-1.6	30.8	20.7	8.5	21.6	5.5	894.8	247.9	0.0	115.7	620.3	5.6	440.7	18.3
	SE	0.5	0.3	0.1	15.8	0.5	0.7	0.3	0.5	0.9	0.5	0.6	27.2	7.2	0.0	2.6	19.2	1.2	25.0	3.4
	*P*	**0.002**	0.167	**0.000**	**0.000**	0.644	**0.000**	**0.000**	**0.000**	**0.000**	0.535	**0.000**	**0.000**	**0.000**	-	**0.000**	**0.000**	**0.000**	**0.000**	**0.000**
**LGM**																				
QLM	Mean	8.1	11.9	3.7	695.7	23.5	-8.9	32.3	16.0	-1.4	16.7	-1.4	541.1	103.6	2.5	80.9	282.4	10.5	273.1	10.5
	SE	1.1	0.6	0.1	22.1	1.3	1.3	0.8	1.2	0.9	1.4	0.9	46.8	8.8	0.5	1.3	23.7	1.8	24.5	1.8
HDM-YGP	Mean	11.7	11.2	4.5	486.6	22.6	-2.3	24.9	17.1	5.1	17.3	4.9	1098.5	229.5	10.6	85.1	618.8	40.1	608.2	40.7
	SE	0.6	0.3	0.1	14.9	0.5	0.8	0.5	0.5	0.8	0.5	0.8	41.6	9.3	1.1	1.7	25.4	3.5	26.9	3.4
	*P*	**0.012**	0.311	**0.001**	**0.000**	0.478	**0.000**	**0.000**	0.358	**0.000**	0.648	**0.000**	**0.000**	**0.000**	**0.000**	0.226	**0.000**	**0.000**	**0.000**	**0.000**
**Current**																				
QLM	Mean	11.9	10.8	3.4	714.7	27.0	-4.7	31.7	19.9	2.2	20.8	2.2	696.6	135.8	3.5	81.5	368.0	14.8	356.8	14.8
	SE	1.3	0.4	0.1	24.8	1.5	1.4	0.5	1.4	1.1	1.6	1.1	55.4	10.0	0.6	1.9	27.5	2.6	28.8	2.6
HDM-YGP	Mean	16.3	10.9	4.4	488.3	27.0	2.5	24.6	21.7	9.6	21.8	9.5	945.8	195.9	9.3	84.3	530.1	35.3	516.3	36.2
	SE	0.5	0.3	0.1	15.2	0.5	0.6	0.4	0.4	0.6	0.4	0.6	19.4	4.7	0.8	1.7	12.6	2.7	15.3	2.7
	*P*	**0.001**	0.879	**0.000**	**0.000**	0.996	**0.000**	**0.000**	0.255	**0.000**	0.52	**0.000**	**0.000**	**0.000**	**0.000**	0.424	**0.000**	**0.000**	**0.000**	**0.000**

Significantly different: P<0.05; significant *P* values were given in bold print.

-, cannot be compared.

**Table 4:** Means, standard errors (SE) and results of independent-Sample t-test on the 19 BioClim factors of the two regions (the QLM, HDM-YGP) for the LIG and LGM periods.

Independent-Sample t-test		bio1	bio2	bio3	bio4	bio5	bio6	bio7	bio8	bio9	bio10	bio11	bio12	bio13	bio14	bio15	bio16	bio17	bio18	bio19
		(°C)	(°C)	(°C)	(SD*100)	(°C)	(°C)	(°C)	(°C)	(°C)	(°C)	(°C)	(mm)	(mm)	(mm)	(mm)	(mm)	(mm)	(mm)	(mm)
**QLM**																				
LIG	Mean	10.4	10.5	2.7	985.1	29.8	-8.4	38.3	14.3	1.0	22.8	-2.8	289.4	162.3	0.0	198.9	231.0	0.0	40.3	0.0
	SE	1.3	0.5	0.2	36.9	1.7	1.3	0.8	1.0	0.9	1.7	0.9	46.7	12.9	0.0	13.4	26.7	0.0	23.0	0.0
LGM	Mean	8.1	11.9	3.7	695.7	23.5	-8.9	32.3	16.0	-1.4	16.7	-1.4	541.1	103.6	2.5	80.9	282.4	10.5	273.1	10.5
	SE	1.1	0.6	0.1	22.1	1.3	1.3	0.8	1.2	0.9	1.4	0.9	46.8	8.8	0.5	1.3	23.7	1.8	24.5	1.8
	***P***	0.186	0.087	**0.001**	**0.000**	**0.010**	0.826	**0.000**	0.302	0.079	**0.015**	0.332	**0.002**	**0.002**	**0.001**	**0.000**	0.172	**0.001**	**0.000**	**0.001**
**HDM-YGP**																				
LIG	Mean	14.3	11.3	3.6	652.5	29.2	-1.6	30.8	20.7	8.5	21.6	5.5	894.8	247.9	0.0	115.7	620.3	5.6	440.7	18.3
	SE	0.5	0.3	0.1	15.8	0.5	0.7	0.3	0.5	0.9	0.5	0.6	27.2	7.2	0.0	2.6	19.2	1.2	25.0	3.4
LGM	Mean	11.7	11.2	4.5	486.6	22.6	-2.3	24.9	17.1	5.1	17.3	4.9	1098.5	229.5	10.6	85.1	618.8	40.1	608.2	40.7
	SE	0.6	0.3	0.1	14.9	0.5	0.8	0.5	0.5	0.8	0.5	0.8	41.6	9.3	1.1	1.7	25.4	3.5	26.9	3.4
	***P***	**0.002**	0.889	**0.000**	**0.000**	**0.000**	0.508	**0.000**	**0.000**	**0.006**	**0.000**	0.539	**0.000**	0.122	**0.000**	**0.000**	0.963	**0.000**	**0.000**	**0.000**

Significantly different: P<0.05; significant *P* values were given in bold print.

**Fig 6:**
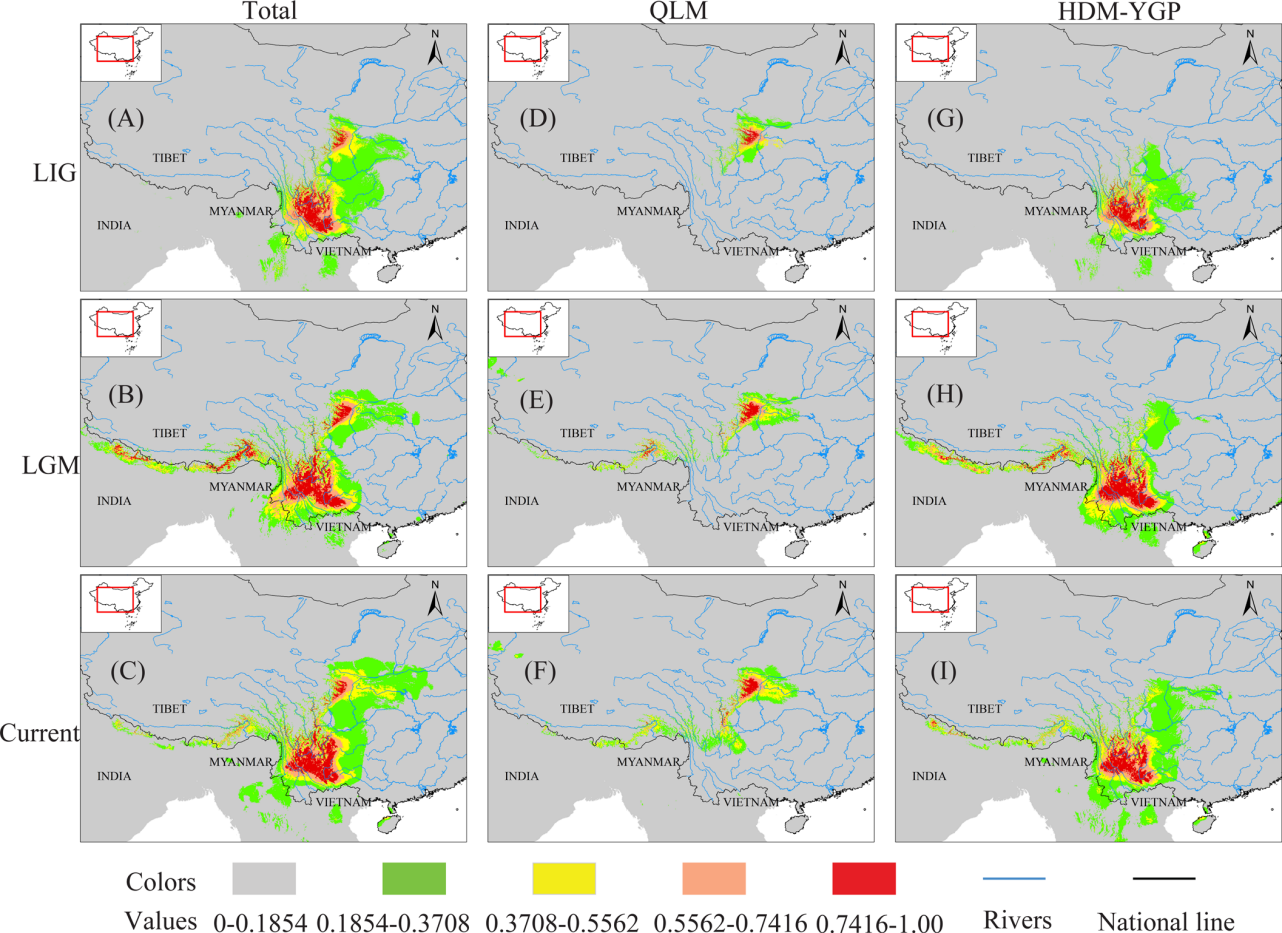
Ecological Niche Modeling (ENM) using bioclimatic factors of the three periods (LIG; LGM; Current) based on the total, QLM, and HDM-YGP populations. Colors with higher values show areas with more suitable predicted conditions. ENM for the total, QLM, and HDM-YGP populations indicated a much wider distribution during the LGM.

The AUCs of the ENM for the total, QLM and HDM-YGP populations under all climate scenarios were ≥ 0.987, indicating quite better model performance of ENM. The current distribution predictions in this study generally represented the actual distributions of *O*. *schwerinae*. Paleo-distribution modeling for the total, QLM and HDM-YGP populations, all indicated a much more restricted distribution during the LIG than the Current, with subsequent expansion during the LGM. This predicted subsequent expansion covered a slightly greater range than that predicted based on the current climatic factors (Fig 6).

## Discussion

### Genetic and Morphological Divergence Relative to Climatic Heterogeneity and Changes

In this study, the SAMOVA, median-joining networks, and Bayesian phylogenetic trees, all suggested population genetic divergence between the QLM and HDM-YGP regions (Figs 2, 3 and 4), which coincide with the morphological divergence of the two varieties of *O*. *schwerinae* in the QLM and HDM-YGP regions. Climatically, the QLM has been primarily dominated by the East Asian monsoon, and is situated near the northern limit of this monsoon system [19] (Fig 1). However, the HDM-YGP has been affected both by the East Asian monsoon from the Pacific and the southwest monsoon from the Indian Ocean [20] (Fig 1). In addition, the HDM-YGP has experienced more intense monsoons than the QLM owing to its more southerly latitude. Therefore, the climate in the HDM-YGP is generally more warm and moist than that in the QLM. Our climate data further confirmed this, demonstrating that the temperature and precipitation values in the QLM were significantly lower than that in the HDM-YGP during the LIG, LGM, and Current periods (Table 3). Considering the two varieties of *O*. *schwerinae*, the leaflet blades of the one growing in the QLM (3–5 mm) are smaller than the one (5–10 mm) growing in the HDM-YGP. Since the sizes and shapes of leaves are quite sensitive to moisture, with leaves in dry climates generally smaller than in wet climates [55], it is quite likely that the difference in morphology of *O*. *schwerinae* between the two regions is the result of long-term adaptation to the significant climatic heterogeneity between the two regions.

It has been speculated that drainage re-organization was a main cause of genetic divergence for riparian plants in the HDM and adjacent regions [16, 17], but we did not detect its effects on *O*. *schwerinae*. When using the same substitution rate range for cpDNA (1.01–3.0×10^−9^ s/s/y) as previous studies on riparian plants [16, 17] in these regions, the genetic divergence time in this study was estimated to be 466.4–159.4 ka (Fig 4A). In addition, the divergence time based on nDNA was calculated to be 315.8–160.3 ka (Fig 4B). They are much more recent than the timings found in previous riparian plants’ studies (4.24–0.4 Ma, *Terminalia franchetii* [17]; 3.683–1.228 Ma, *Buddleja crispa* [16]) and the geological drainage re-organization events (no later than the later Pliocene; 5.332–2.588 Ma) [56–58]. However, our divergence times largely coincide with the time of the weakening of the monsoons in these regions. According to paleo-climatic analysis, the southwest monsoon was sharply weakened from 342–118 ka [59]. Coincidentally, the East Asian monsoon was also found to decrease during the period 200–130 ka [23, 27, 60]. Because of this, weaker airflows associated with monsoons would reach the HDM-YGP as well as the QLM, which probably led to a decrease in precipitation in both regions. On one hand, reduced precipitation from 342-118/200-130 ka would cause the contraction of the *O*. *schwerinae* populations in both regions (e.g. Fig 6A, 6D and 6G), and result in the loss/reduction of gene flow between them. On the other hand, the weakening of monsoons probably further increased the climatic differences between the two regions. For instance, the LIG (c. 140–120 ka) falls in the aforementioned time range of the weakening of monsoons (342-118/200-130 ka), and, as expected, displayed two more significantly different bioclimatic factors between the QLM and HDM-YGP than the LGM and the Current periods (Table 3). Finally, these two reasons together led to the genetic divergence between the QLM and HDM-YGP regions.

### Demographic History of *Osteomeles schwerinae*

Usually, the demographic scenarios reported for species from the HDM and adjacent regions suggest distribution contraction during glacial periods, and expansions during interglacial periods [8–11]. However, our results revealed an unusual demographic history of *O*. *schwerinae* populations: the opposite pattern to most demographic scenarios in the HDM and adjacent regions. Before the period “342-118/200-130 ka” (including the LIG; c. 140–120 ka), the *O*. *schwerinae* populations probably had a more continuous or wider distribution linking the two geographic regions because of stronger monsoons/more precipitation in both regions. However, during the LIG, the *O*. *schwerinae* populations probably had a less continuous or more narrow distribution because of weaker monsoons/less precipitation. The ENM analysis further confirmed this, showing more narrow distribution during the LIG, but larger distribution ranges during the LGM period (Fig 6), which implied the “contraction during interglacial periods, and expansions during glacial periods” demographic pattern of *O*. *schwerinae* populations. Unimodal pairwise mismatch distribution curves of *O*. *schwerinae* (Fig 5) were identified in this study, which fit the sudden expansion model particularly well. Moreover, the expansion times for the total, QLM and HDM-YGP populations of *O*. *schwerinae* fell into the last glacial period (Table 2), which further supported this pattern.

This pattern is similar to the “phalanx” model [61], which has only been sporadically found in a few cold-adapted species at high-elevation in (sub)tropical areas [62–65]. These plants were found to expand to lower elevations during glacial periods, allowing isolated patches to join together, because of the retreat of the (sub)tropical forests occupying lower altitudes. In contrast, these plants would be forced back to ‘refugia’ at higher elevations during interglacial periods as a result of the re-expansion of the (sub)tropical forests at low elevations [66]. In the HDM and adjacent regions, the majority of plants have been found to retreat to refugia because of cooling during the glacial periods, which probably left a large space for *O*. *schwerinae* populations to expand during the last glacial period. Meanwhile, because of the Foehn effect [67, 68], the climate in river valleys is warmer than in neighboring areas [15]. Therefore, the cooling during the last glacial period might have had a limited impact on the populations of *O*. *schwerinae*. For instance, the annual global-mean continental cooling during the LGM has been found to have been 4.3–9.8°C [69, 70]. In contrast, the climatic data for our sampling sites reveals a lower annual average temperature reduction during the LGM (only 2.3 and 2.6°C lower than the LIG for the QLM and HDM-YGP, respectively; Table 4), which probably reflects the warm climate in river valleys. In addition, owing to the intensification of the Asian monsoons affecting precipitation during the last glacial period in these regions [23, 27, 28, 59], the precipitation at our sampling sites was significantly increased during the LGM compared to the LIG (Table 4). This may have relieved or even eliminated the impact of the drought since 342-118/200-130 ka [23, 27, 59, 60].

In this context, the river valleys could have provided warm and moist habits for *O*. *schwerinae* to expand during the last glacial period, resulting in a second contact between the populations from the QLM and HDM-YGP. This second contact probably just explains the high cpDNA and nDNA haplotype diversity within their intersecting distributions in the Dadu River basin, where there is the common haplotype (cpDNA: H4; nDNA: Hap3), regional haplotypes both from the QLM (cpDNA: H1; nDNA: Hap2, 5, 11) and HDM-YGP (cpDNA: H2; nDNA: Hap7), as well as its own exclusive haplotypes (cpDNA: H5; nDNA: Hap12) (Table 1; Fig 2A and 2B).

## Conclusions

Our analyses of *O*. *schwerinae*, based on molecular phylogeography and climatic data, reveal a major population divergence between the QLM and HDM-YGP regions, and its correlation to the climatic heterogeneity and changes within both regions since the late Pleistocene. We inferred that weakening of Asian monsoons since 342-118/200-130 ka increased the climatic differences between the two regions, caused the loss/reduction of gene flow, and triggered the genetic divergence between them. Subsequent intensification of the monsoons during the last glaciation facilitated the expansion of *O*. *schwerinae* populations, contributed to the second contact between the populations from the QLM and HDM-YGP, and finally led to the development of the current distribution pattern of *O*. *schwerinae* populations. This study provides a new example in which riparian plant species in the HDM and adjacent regions are shown to be affected by climatic heterogeneity and changes since the late Pleistocene. However, more studies of riparian plants in these regions are needed to explore this hypothesis.

## Supporting Information

S1 Fig Distributions of the *F*_CT_ values for indicated numbers of groups (*K*) of *Osteomeles schwerinae* populations based on cpDNA sequences. (TIF)

S2 Fig Distributions of the *F*_CT_ values for indicated numbers of groups (*K*) of *Osteomeles schwerinae* populations based on nDNA sequences. (TIF)

S1 Table BioClim factors used to define the ecological habitats of 40 *Osteomeles schwerinae* populations. (DOCX)

S2 Table BioClim factors for 40 *Osteomeles schwerinae* populations during the LIG, LGM, and Current periods. (DOCX)

S3 Table Information on the collection records obtained from the three main herbaria in China (IBSC, KUN and PE). (DOCX)

S4 Table Polymorphic sites in the three aligned cpDNA sequences, and defining features (substitutions and indels) of the six cpDNA haplotypes (H1-6). (DOCX)

S5 Table Polymorphic sites in the aligned nDNA sequences, and defining features (substitutions) of the 15 nDNA haplotypes (Hap1-15). (DOCX)
