# Supplementary figures and images for: Climatic Factors Drive Population Divergence and Demography: Insights Based on the Phylogeography of a Riparian Plant Species Endemic to the Hengduan Mountains and Adjacent Regions
Source: PLoS One. 2015 Dec 21;10(12):e0145014. doi: 10.1371/journal.pone.0145014 (PMC4687034; doi:10.1371/journal.pone.0145014)

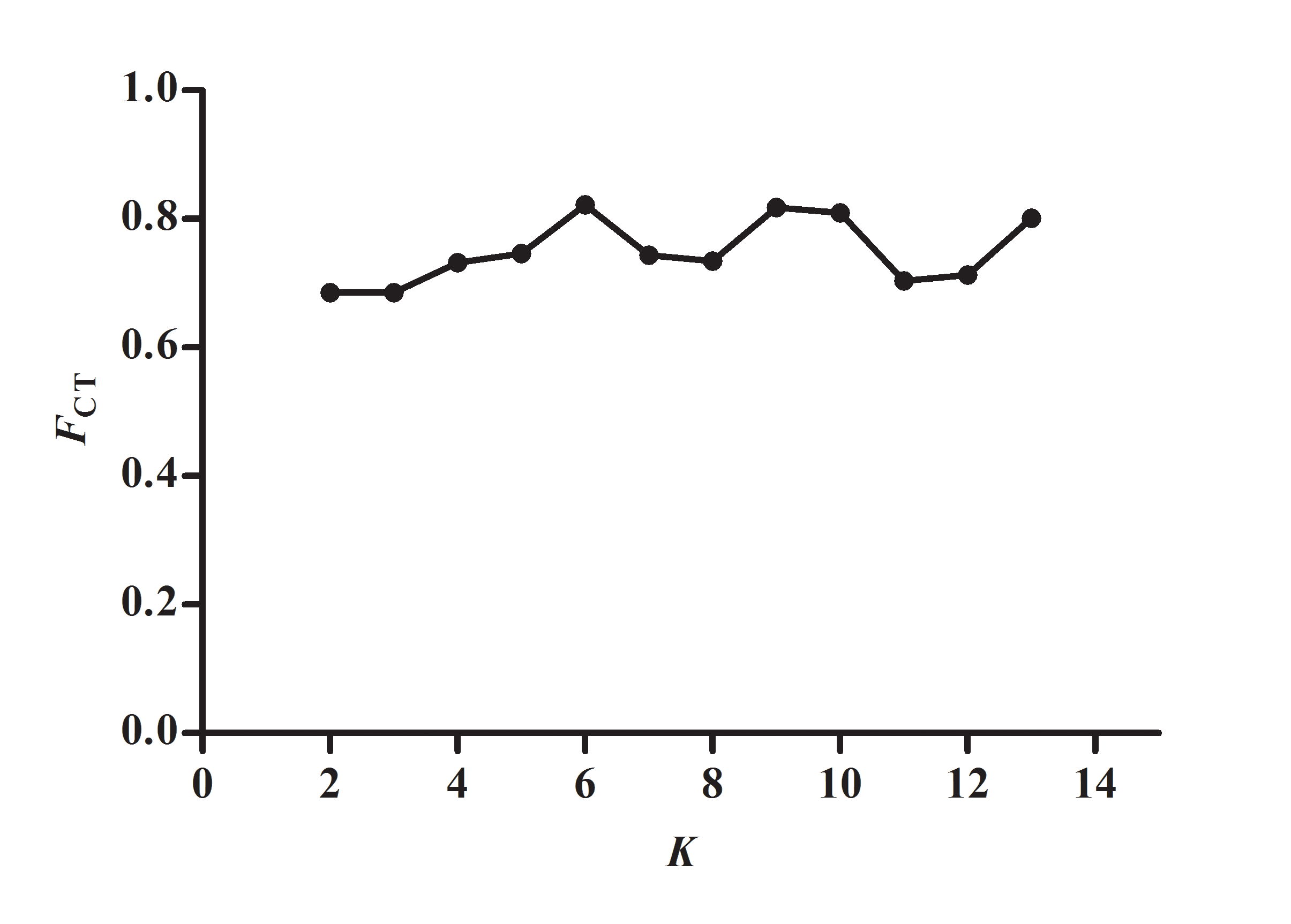

Supplement: S1 Fig — (TIF) [file pone.0145014.s001.tif]

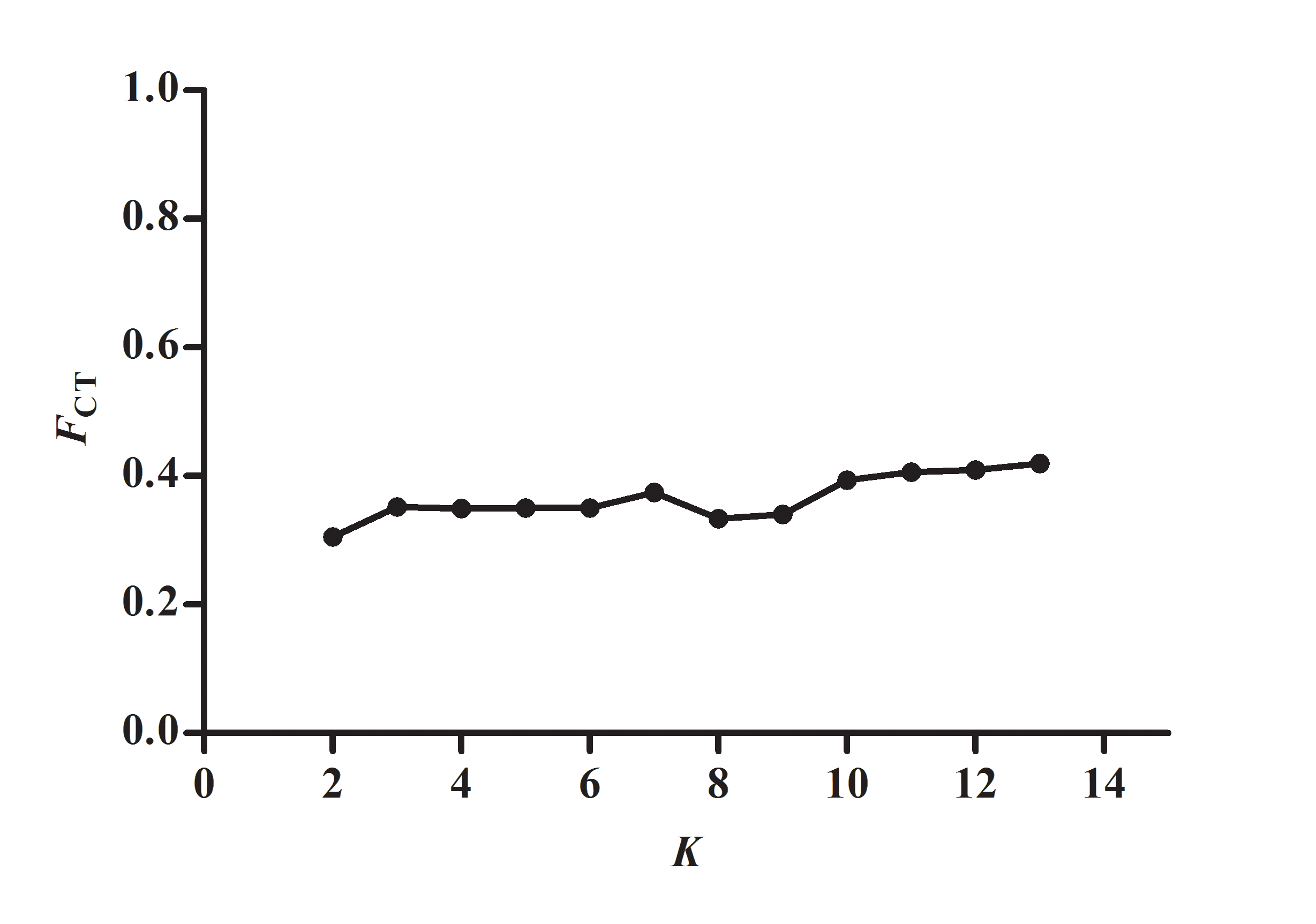

Supplement: S2 Fig — (TIF) [file pone.0145014.s002.tif]
