# Supplementary material for: Climatic Factors Drive Population Divergence and Demography: Insights Based on the Phylogeography of a Riparian Plant Species Endemic to the Hengduan Mountains and Adjacent Regions
Source: PLoS One. 2015 Dec 21;10(12):e0145014. doi: 10.1371/journal.pone.0145014 (PMC4687034; doi:10.1371/journal.pone.0145014)
Supplement: S1 Table — (DOCX) [file pone.0145014.s003.docx]

| BioClim factors | What do they represent? |
| --- | --- |
| bio1 | Annual Mean Temperature |
| bio2 | Mean Diurnal Range (Mean of monthly (max temp - min temp) |
| bio3 | Isothermality (BIO2/BIO7) (* 100) |
| bio4 | Temperature Seasonality (standard deviation *100) |
| bio5 | Max Temperature of Warmest Month |
| bio6 | Min Temperature of Coldest Month |
| bio7 | Temperature Annual Range (BIO5-BIO6) |
| bio8 | Mean Temperature of Wettest Quarter |
| bio9 | Mean Temperature of Driest Quarter |
| bio10 | Mean Temperature of Warmest Quarter |
| bio11 | Mean Temperature of Coldest Quarter |
| bio12 | Annual Precipitation |
| bio13 | Precipitation of Wettest Month |
| bio14 | Precipitation of Driest Month |
| bio15 | Precipitation Seasonality (Coefficient of Variation) |
| bio16 | Precipitation of Wettest Quarter |
| bio17 | Precipitation of Driest Quarter |
| bio18 | Precipitation of Warmest Quarter |
| bio19 | Precipitation of Coldest Quarter |
