# Supplementary material for: Climatic Factors Drive Population Divergence and Demography: Insights Based on the Phylogeography of a Riparian Plant Species Endemic to the Hengduan Mountains and Adjacent Regions
Source: PLoS One. 2015 Dec 21;10(12):e0145014. doi: 10.1371/journal.pone.0145014 (PMC4687034; doi:10.1371/journal.pone.0145014)
Supplement: S2 Table — (DOCX) [file pone.0145014.s004.docx]

| Periods | Regions/Populations | bio1 | bio2 | bio3 | bio4 | bio5 | bio6 | bio7 | bio8 | bio9 | bio10 | bio11 | bio12 | bio13 | bio14 | bio15 | bio16 | bio17 | bio18 | bio19 |
| --- | --- | --- | --- | --- | --- | --- | --- | --- | --- | --- | --- | --- | --- | --- | --- | --- | --- | --- | --- | --- |
|  |  | (℃) | (℃) | (℃) | (SD*100) | (℃) | (℃) | (℃) | (℃) | (℃) | (℃) | (℃) | (mm) | (mm) | (mm) | (mm) | (mm) | (mm) | (mm) | (mm) |
| LIG | QLM region |  |  |  |  |  |  |  |  |  |  |  |  |  |  |  |  |  |  |  |
|  | 1 | 10.4 | 11.2 | 2.7 | 1022.2 | 30.8 | -9.5 | 40.3 | 13.2 | 0.5 | 23.4 | -3.1 | 208 | 147 | 0 | 228 | 192 | 0 | 0 | 0 |
|  | 2 | 7.7 | 10.4 | 2.6 | 1015.1 | 28.1 | -11.8 | 39.9 | 10.3 | -2.2 | 20.8 | -5.7 | 223 | 149 | 0 | 221 | 199 | 0 | 0 | 0 |
|  | 3 | 14.3 | 9.1 | 2.3 | 1050.9 | 34.8 | -4.4 | 39.2 | 17.3 | 3.6 | 27.6 | 0.2 | 185 | 133 | 0 | 233 | 175 | 0 | 0 | 0 |
|  | 4 | 14.6 | 9.1 | 2.4 | 1022.2 | 34 | -3.4 | 37.4 | 17.6 | 3.9 | 27.3 | 0.8 | 249 | 145 | 0 | 193 | 189 | 0 | 35 | 0 |
|  | 5 | 13.7 | 8.8 | 2.1 | 1134.3 | 34.9 | -5.8 | 40.7 | 16.8 | 2.1 | 27.9 | -1.8 | 223 | 126 | 0 | 195 | 181 | 0 | 0 | 0 |
|  | 6 | 10 | 11.4 | 2.9 | 967.2 | 29.2 | -9.3 | 38.5 | 12.8 | 0.4 | 22.2 | -2.9 | 224 | 160 | 0 | 235 | 208 | 0 | 0 | 0 |
|  | 7 | 4 | 12.5 | 3.5 | 831.1 | 21.1 | -14.6 | 35.7 | 10.6 | -2.5 | 14.2 | -7.1 | 510 | 223 | 0 | 144 | 363 | 0 | 136 | 0 |
|  | 8 | 8.8 | 11.2 | 3.2 | 837.5 | 25.8 | -8.7 | 34.5 | 15.7 | 2.1 | 18.9 | -2.4 | 493 | 215 | 0 | 142 | 341 | 0 | 151 | 0 |
|  | HDM-YGP region |  |  |  |  |  |  |  |  |  |  |  |  |  |  |  |  |  |  |  |
|  | 9 | 11.2 | 11.9 | 3.2 | 822.9 | 29 | -7.1 | 36.1 | 18 | 4.2 | 20.8 | 0.4 | 470 | 198 | 0 | 155 | 378 | 0 | 176 | 0 |
|  | 10 | 13.8 | 9.4 | 2.8 | 792.2 | 30.3 | -2.3 | 32.6 | 22.5 | 7.3 | 22.8 | 3.2 | 713 | 198 | 0 | 113 | 433 | 0 | 374 | 0 |
|  | 11 | 10 | 10.4 | 3 | 814.3 | 26.8 | -7.3 | 34.1 | 16.7 | 3.1 | 19.4 | -0.8 | 433 | 203 | 0 | 165 | 370 | 0 | 141 | 0 |
|  | 12 | 11.8 | 13 | 4.1 | 627.1 | 25.8 | -5.3 | 31.1 | 18 | 6.5 | 18.6 | 3.4 | 879 | 255 | 0 | 121 | 617 | 0 | 583 | 0 |
|  | 13 | 15.8 | 12.7 | 3.9 | 629.3 | 30.5 | -1.3 | 31.8 | 21.8 | 14.9 | 22.8 | 7.4 | 930 | 254 | 0 | 125 | 691 | 0 | 598 | 2 |
|  | 14 | 11.3 | 12.9 | 3.8 | 696.6 | 27.2 | -6.4 | 33.6 | 18.5 | 2.4 | 18.7 | 1.9 | 880 | 260 | 0 | 112 | 571 | 3 | 544 | 3 |
|  | 15 | 13.3 | 12.3 | 3.9 | 650.7 | 27.6 | -3.7 | 31.3 | 19.8 | 5 | 20 | 4.4 | 1061 | 283 | 0 | 102 | 670 | 8 | 616 | 15 |
|  | 16 | 11.5 | 13.4 | 4 | 669.7 | 26.8 | -6.4 | 33.2 | 18.3 | 6 | 18.6 | 2.5 | 779 | 235 | 0 | 120 | 525 | 0 | 509 | 0 |
|  | 17 | 11.6 | 12.7 | 4.1 | 621 | 25.2 | -5.1 | 30.3 | 17.9 | 3.5 | 18.3 | 3.3 | 883 | 247 | 0 | 119 | 626 | 0 | 564 | 0 |
|  | 18 | 12.7 | 12.3 | 4.1 | 593.8 | 26.2 | -3.3 | 29.5 | 18.6 | 4.9 | 19.3 | 4.7 | 933 | 247 | 0 | 120 | 672 | 4 | 574 | 4 |
|  | 19 | 17.6 | 12.8 | 4 | 607.8 | 32.3 | 0.9 | 31.4 | 23.1 | 17.4 | 24.4 | 9.3 | 773 | 211 | 0 | 123 | 576 | 0 | 488 | 11 |
|  | 20 | 17.2 | 12.3 | 4 | 591 | 31.2 | 1.1 | 30.1 | 22.9 | 9.3 | 23.8 | 9.3 | 830 | 206 | 0 | 111 | 580 | 10 | 483 | 23 |
|  | 21 | 14.3 | 12.5 | 3.9 | 631.9 | 29.2 | -2.7 | 31.9 | 20.4 | 13.3 | 21.4 | 5.9 | 972 | 266 | 0 | 125 | 729 | 0 | 631 | 2 |
|  | 22 | 12.6 | 11.8 | 3.8 | 608 | 27.2 | -3.4 | 30.6 | 18.4 | 11.9 | 19.4 | 4.5 | 876 | 266 | 0 | 127 | 669 | 0 | 586 | 12 |
|  | 23 | 13.1 | 9 | 2.9 | 783.1 | 28.3 | -2.6 | 30.9 | 21.6 | 7.6 | 21.6 | 2.3 | 778 | 171 | 0 | 113 | 506 | 0 | 506 | 0 |
|  | 24 | 13.9 | 7.8 | 2.4 | 857.3 | 30.2 | -1.9 | 32.1 | 23.6 | 1.9 | 23.6 | 1.9 | 782 | 167 | 0 | 93 | 452 | 5 | 452 | 5 |
|  | 25 | 16 | 8 | 2.4 | 860 | 32.7 | 0.2 | 32.5 | 25.7 | 4 | 25.7 | 4 | 762 | 173 | 0 | 93 | 445 | 5 | 445 | 5 |
|  | 26 | 13.5 | 11.7 | 4.1 | 577.9 | 26.5 | -1.9 | 28.4 | 19.4 | 5.6 | 19.9 | 5.6 | 1011 | 232 | 0 | 102 | 660 | 22 | 525 | 22 |
|  | 27 | 14.1 | 11.8 | 4.1 | 586.5 | 26.9 | -1.8 | 28.7 | 20.2 | 6 | 20.6 | 6 | 1005 | 215 | 0 | 94 | 623 | 31 | 500 | 31 |
|  | 28 | 11.9 | 11.6 | 4.1 | 563.4 | 24.6 | -3.5 | 28.1 | 17.9 | 4.2 | 18.3 | 4.2 | 1032 | 237 | 0 | 105 | 693 | 17 | 539 | 17 |
|  | 29 | 18.9 | 11.5 | 3.9 | 580 | 33.3 | 4 | 29.3 | 24.3 | 18.7 | 25.6 | 11.2 | 954 | 267 | 0 | 115 | 689 | 4 | 288 | 50 |
|  | 30 | 22.2 | 11.6 | 3.9 | 583 | 37 | 7.4 | 29.6 | 27.5 | 14.5 | 29 | 14.4 | 918 | 246 | 0 | 109 | 637 | 13 | 295 | 59 |
|  | 31 | 17.6 | 11.2 | 3.8 | 585.9 | 32.6 | 3.3 | 29.3 | 23 | 17.6 | 24.6 | 9.9 | 981 | 292 | 0 | 121 | 732 | 3 | 297 | 44 |
|  | 32 | 17.4 | 10.5 | 3.6 | 575.3 | 32.4 | 3.9 | 28.5 | 22.3 | 10.1 | 24.4 | 9.7 | 963 | 293 | 0 | 116 | 694 | 6 | 288 | 48 |
|  | 33 | 14.5 | 10.8 | 3.5 | 604.8 | 29.5 | -0.6 | 30.1 | 20 | 14.1 | 21.4 | 6.5 | 975 | 276 | 0 | 117 | 712 | 4 | 314 | 35 |
|  | 34 | 15.7 | 11 | 3.6 | 612.3 | 31.3 | 0.8 | 30.5 | 21.3 | 15.5 | 22.8 | 7.6 | 934 | 271 | 0 | 119 | 685 | 7 | 302 | 38 |
|  | 35 | 15.6 | 11 | 3.8 | 571.5 | 29.7 | 1.1 | 28.6 | 21.1 | 15.3 | 22.2 | 8.1 | 1042 | 298 | 0 | 116 | 753 | 6 | 307 | 49 |
|  | 36 | 16.2 | 10.3 | 3.5 | 599.8 | 31.6 | 2.3 | 29.3 | 21.5 | 8.5 | 23.4 | 8.3 | 946 | 293 | 0 | 119 | 695 | 5 | 291 | 43 |
|  | 37 | 16.4 | 8.7 | 3.1 | 608.2 | 31.3 | 3.4 | 27.9 | 21.9 | 8.3 | 23.5 | 8.3 | 1049 | 308 | 0 | 111 | 730 | 4 | 335 | 4 |
|  | 38 | 14.9 | 9.5 | 3.2 | 618.4 | 30.4 | 1.2 | 29.2 | 20.5 | 6.8 | 22.2 | 6.8 | 1025 | 297 | 0 | 112 | 720 | 5 | 332 | 40 |
|  | 39 | 9.4 | 12.2 | 3.7 | 690.2 | 25 | -7.9 | 32.9 | 16.6 | 0.8 | 16.6 | 0 | 931 | 266 | 0 | 108 | 597 | 7 | 563 | 7 |
|  | 40 | 11.8 | 12.2 | 3.8 | 666.9 | 26.4 | -5.4 | 31.8 | 18.5 | 3.4 | 18.7 | 2.6 | 1133 | 301 | 0 | 102 | 718 | 9 | 655 | 17 |
| LGM | QLM region |  |  |  |  |  |  |  |  |  |  |  |  |  |  |  |  |  |  |  |
|  | 1 | 7.3 | 14.3 | 4 | 718.1 | 23.9 | -11.5 | 35.4 | 15.4 | -2.6 | 16.1 | -2.6 | 415 | 78 | 1 | 79 | 210 | 6 | 201 | 6 |
|  | 2 | 5.3 | 13.5 | 3.8 | 719.3 | 21.9 | -12.9 | 34.8 | 13.5 | -4.4 | 14.3 | -4.4 | 450 | 82 | 2 | 79 | 228 | 8 | 218 | 8 |
|  | 3 | 12.1 | 10.6 | 3.3 | 737.1 | 27.6 | -4.4 | 32 | 20.1 | 2.1 | 21.4 | 2.1 | 419 | 83 | 1 | 85 | 229 | 5 | 218 | 5 |
|  | 4 | 11 | 9.8 | 3.2 | 701.9 | 25.8 | -4.5 | 30.3 | 18.8 | 1.5 | 19.8 | 1.5 | 527 | 112 | 2 | 87 | 298 | 9 | 286 | 9 |
|  | 5 | 11.7 | 9.9 | 3 | 792.1 | 28.1 | -4.6 | 32.7 | 20.5 | 1 | 21.7 | 1 | 610 | 118 | 4 | 83 | 338 | 13 | 315 | 13 |
|  | 6 | 6.7 | 13.9 | 4.1 | 676.7 | 22.6 | -11.3 | 33.9 | 14.4 | -2.5 | 15 | -2.5 | 451 | 84 | 2 | 78 | 225 | 8 | 216 | 8 |
|  | 7 | 3.5 | 12.1 | 4 | 602.6 | 17.4 | -12.8 | 30.2 | 11 | -4.7 | 11 | -4.7 | 738 | 133 | 4 | 76 | 363 | 19 | 363 | 19 |
|  | 8 | 6.8 | 11.3 | 3.8 | 617.6 | 20.5 | -8.9 | 29.4 | 14.3 | -1.7 | 14.3 | -1.7 | 719 | 139 | 4 | 80 | 368 | 16 | 368 | 16 |
|  | HDM-YGP region |  |  |  |  |  |  |  |  |  |  |  |  |  |  |  |  |  |  |  |
|  | 9 | 7.1 | 13.2 | 4.2 | 605.5 | 21.2 | -9.8 | 31 | 13 | -1.3 | 14.2 | -1.3 | 551 | 118 | 2 | 87 | 280 | 9 | 279 | 9 |
|  | 10 | 7.8 | 9.1 | 3.4 | 597.2 | 20.4 | -5.7 | 26.1 | 15 | -0.5 | 15 | -0.5 | 797 | 140 | 4 | 79 | 402 | 19 | 402 | 19 |
|  | 11 | 6.3 | 11.5 | 3.9 | 602 | 19.9 | -9.5 | 29.4 | 12.2 | -2 | 13.5 | -2 | 597 | 123 | 2 | 83 | 299 | 11 | 291 | 11 |
|  | 12 | 10.2 | 13.2 | 5 | 470.7 | 21.1 | -5 | 26.1 | 15.7 | 3.7 | 15.7 | 3.7 | 1141 | 262 | 3 | 99 | 701 | 14 | 701 | 14 |
|  | 13 | 12.1 | 14.2 | 5 | 473.6 | 23.8 | -4.1 | 27.9 | 17.3 | 5.4 | 17.3 | 5.4 | 1406 | 328 | 9 | 101 | 885 | 27 | 885 | 27 |
|  | 14 | 5.6 | 11.1 | 4.3 | 525.6 | 16.9 | -8.4 | 25.3 | 11.9 | -0.3 | 11.9 | -1.4 | 725 | 164 | 6 | 86 | 418 | 24 | 418 | 25 |
|  | 15 | 10.3 | 11.1 | 4.6 | 484 | 20.5 | -3.6 | 24.1 | 16 | 4.6 | 16 | 3.7 | 1117 | 244 | 7 | 86 | 645 | 32 | 645 | 38 |
|  | 16 | 7.2 | 13 | 4.8 | 513.3 | 18.5 | -8.5 | 27 | 13.2 | 0.2 | 13.2 | 0.2 | 856 | 208 | 3 | 99 | 535 | 14 | 535 | 14 |
|  | 17 | 11.5 | 12.3 | 4.9 | 464.8 | 21.9 | -3.1 | 25 | 16.8 | 5.1 | 16.8 | 5.1 | 1117 | 251 | 4 | 94 | 673 | 24 | 673 | 24 |
|  | 18 | 11.1 | 12.5 | 5 | 439.8 | 21.5 | -3.2 | 24.7 | 16.1 | 5 | 16.1 | 5 | 1220 | 276 | 6 | 99 | 755 | 25 | 755 | 25 |
|  | 19 | 16.8 | 14 | 5.1 | 451.7 | 28.4 | 1.1 | 27.3 | 21.4 | 10.4 | 21.6 | 10.4 | 973 | 215 | 8 | 92 | 576 | 27 | 458 | 27 |
|  | 20 | 13.5 | 12.5 | 5 | 429.5 | 24.1 | -0.7 | 24.8 | 18.3 | 7.5 | 18.3 | 7.5 | 1144 | 235 | 11 | 91 | 671 | 38 | 671 | 38 |
|  | 21 | 11.6 | 14.4 | 5 | 485.2 | 23.5 | -4.8 | 28.3 | 17 | 4.7 | 17 | 4.7 | 1421 | 333 | 9 | 100 | 892 | 27 | 892 | 27 |
|  | 22 | 9.2 | 13.5 | 5.1 | 453.6 | 20.1 | -5.9 | 26 | 14.3 | 2.8 | 14.3 | 2.8 | 1330 | 333 | 9 | 96 | 823 | 40 | 823 | 40 |
|  | 23 | 9.9 | 9.2 | 3.2 | 644.3 | 24.1 | -4.2 | 28.3 | 17.5 | 0.9 | 17.5 | 0.9 | 1264 | 256 | 15 | 80 | 689 | 53 | 689 | 53 |
|  | 24 | 8.9 | 7.7 | 2.7 | 697.2 | 23.4 | -4.3 | 27.7 | 17.5 | -0.6 | 17.5 | -0.6 | 1514 | 240 | 28 | 64 | 696 | 89 | 696 | 89 |
|  | 25 | 9.8 | 8 | 2.8 | 703.4 | 24.6 | -3.5 | 28.1 | 16.8 | 0.3 | 18.5 | 0.3 | 1513 | 245 | 27 | 64 | 693 | 89 | 689 | 89 |
|  | 26 | 11.1 | 11.2 | 4.9 | 423.2 | 20.7 | -2 | 22.7 | 16 | 5.3 | 16 | 5.3 | 1247 | 264 | 14 | 87 | 705 | 55 | 705 | 55 |
|  | 27 | 13.1 | 11 | 4.8 | 433 | 22.3 | -0.5 | 22.8 | 18 | 7 | 18 | 7 | 1158 | 225 | 17 | 76 | 605 | 69 | 605 | 69 |
|  | 28 | 10.7 | 11.1 | 4.9 | 413.5 | 20 | -2.6 | 22.6 | 15.4 | 4.9 | 15.4 | 4.9 | 1205 | 257 | 15 | 87 | 685 | 52 | 685 | 52 |
|  | 29 | 17.4 | 10.9 | 4.8 | 410.4 | 26.8 | 4.4 | 22.4 | 21.8 | 13.3 | 21.8 | 11.6 | 1037 | 206 | 13 | 82 | 584 | 45 | 584 | 45 |
|  | 30 | 21.3 | 10.7 | 4.8 | 408.4 | 30.9 | 8.7 | 22.2 | 25.5 | 15.4 | 25.6 | 15.4 | 894 | 157 | 11 | 71 | 455 | 47 | 405 | 47 |
|  | 31 | 15.7 | 10.6 | 4.8 | 412.9 | 25 | 3 | 22 | 20.1 | 9.8 | 20.1 | 9.8 | 1073 | 224 | 11 | 85 | 621 | 44 | 621 | 44 |
|  | 32 | 15.6 | 9.9 | 4.7 | 398.7 | 24.7 | 3.8 | 20.9 | 19.8 | 9.9 | 19.8 | 9.9 | 1007 | 209 | 10 | 80 | 551 | 45 | 472 | 45 |
|  | 33 | 13 | 11.5 | 4.7 | 447.1 | 23 | -1 | 24 | 17.9 | 6.6 | 17.9 | 6.6 | 1229 | 252 | 15 | 85 | 709 | 54 | 709 | 54 |
|  | 34 | 14.2 | 11.4 | 4.7 | 448.8 | 24.3 | 0.4 | 23.9 | 19 | 7.7 | 19 | 7.7 | 1115 | 225 | 13 | 83 | 633 | 51 | 633 | 51 |
|  | 35 | 13.8 | 10.5 | 4.7 | 405.6 | 23 | 1 | 22 | 18.3 | 8.1 | 18.3 | 8.1 | 1168 | 244 | 15 | 85 | 674 | 49 | 674 | 49 |
|  | 36 | 14.8 | 10.3 | 4.7 | 423.3 | 24.1 | 2.3 | 21.8 | 19.2 | 8.7 | 19.2 | 8.7 | 1015 | 214 | 10 | 81 | 566 | 45 | 486 | 45 |
|  | 37 | 14.5 | 8.8 | 4.1 | 450.7 | 23.7 | 2.7 | 21 | 19.3 | 7.9 | 19.3 | 7.9 | 1108 | 224 | 12 | 78 | 595 | 45 | 595 | 45 |
|  | 38 | 13.1 | 9.8 | 4.4 | 457.6 | 22.5 | 0.4 | 22.1 | 18.1 | 6.5 | 18.1 | 6.5 | 1123 | 222 | 15 | 78 | 607 | 51 | 607 | 51 |
|  | 39 | 6.6 | 10.3 | 4.2 | 510.9 | 17.4 | -6.7 | 24.1 | 12.7 | 0.8 | 12.7 | -0.2 | 835 | 183 | 7 | 82 | 466 | 30 | 466 | 33 |
|  | 40 | 11 | 10.5 | 4.4 | 486.7 | 21 | -2.7 | 23.7 | 16.7 | 5.3 | 16.7 | 4.3 | 1253 | 266 | 9 | 83 | 712 | 38 | 712 | 49 |
| Current | QLM region |  |  |  |  |  |  |  |  |  |  |  |  |  |  |  |  |  |  |  |
|  | 1 | 11.6 | 11.9 | 3.5 | 755 | 27.6 | -6.3 | 33.9 | 19.9 | 1.2 | 20.8 | 1.2 | 543 | 104 | 2 | 79 | 277 | 9 | 264 | 9 |
|  | 2 | 8.9 | 11.1 | 3.3 | 736 | 24.4 | -8.3 | 32.7 | 17.2 | -1.1 | 18 | -1.1 | 603 | 110 | 3 | 78 | 304 | 12 | 291 | 12 |
|  | 3 | 15.8 | 9.6 | 3 | 756.4 | 31.2 | -0.4 | 31.6 | 24 | 5.5 | 25.3 | 5.5 | 562 | 114 | 2 | 86 | 313 | 9 | 301 | 9 |
|  | 4 | 16.2 | 9.4 | 3 | 736.2 | 31.3 | 0.5 | 30.8 | 24.2 | 6.2 | 25.4 | 6.2 | 700 | 157 | 3 | 92 | 417 | 11 | 405 | 11 |
|  | 5 | 15.2 | 9.2 | 2.8 | 805.8 | 31.3 | -1 | 32.3 | 24.1 | 4.2 | 25.3 | 4.2 | 735 | 143 | 4 | 83 | 409 | 16 | 383 | 16 |
|  | 6 | 11.4 | 11.9 | 3.6 | 706.8 | 26.7 | -5.9 | 32.6 | 19.3 | 1.7 | 20 | 1.7 | 580 | 115 | 2 | 79 | 298 | 10 | 285 | 10 |
|  | 7 | 5.7 | 12.4 | 4 | 602.4 | 19.6 | -10.9 | 30.5 | 13.1 | -2.5 | 13.1 | -2.5 | 958 | 169 | 7 | 75 | 467 | 29 | 467 | 29 |
|  | 8 | 10.6 | 11 | 3.7 | 618.8 | 24.2 | -4.9 | 29.1 | 17.6 | 2 | 18.1 | 2 | 892 | 174 | 5 | 80 | 459 | 22 | 458 | 22 |
|  | HDM-YGP region |  |  |  |  |  |  |  |  |  |  |  |  |  |  |  |  |  |  |  |
|  | 9 | 13.1 | 12 | 4 | 620.8 | 27 | -2.9 | 29.9 | 20.4 | 4.5 | 20.4 | 4.5 | 661 | 131 | 3 | 83 | 331 | 12 | 331 | 12 |
|  | 10 | 15.7 | 9.4 | 3.5 | 616 | 28.5 | 1.7 | 26.8 | 22.6 | 7.2 | 23.1 | 7.2 | 923 | 190 | 5 | 86 | 513 | 21 | 498 | 21 |
|  | 11 | 11.9 | 10.3 | 3.6 | 612.6 | 25.1 | -3.2 | 28.3 | 17.9 | 3.3 | 19.1 | 3.3 | 646 | 132 | 3 | 82 | 324 | 13 | 306 | 13 |
|  | 12 | 13.8 | 12.6 | 4.9 | 474.6 | 24.3 | -1.3 | 25.6 | 19.2 | 7.2 | 19.2 | 7.2 | 933 | 215 | 3 | 100 | 574 | 13 | 574 | 13 |
|  | 13 | 17.7 | 12.4 | 4.7 | 479.6 | 28.7 | 2.4 | 26.3 | 23 | 11 | 23 | 11 | 968 | 220 | 6 | 99 | 599 | 20 | 599 | 20 |
|  | 14 | 13.5 | 12.1 | 4.6 | 517.6 | 24.9 | -1.1 | 26 | 19.6 | 7.4 | 19.6 | 6.5 | 893 | 198 | 5 | 88 | 520 | 23 | 520 | 27 |
|  | 15 | 15.5 | 11.5 | 4.6 | 481.8 | 25.8 | 1.3 | 24.5 | 21.1 | 9.7 | 21.1 | 8.9 | 1094 | 227 | 9 | 82 | 613 | 36 | 613 | 45 |
|  | 16 | 13.6 | 12.8 | 4.8 | 506 | 24.8 | -1.8 | 26.6 | 19.5 | 6.6 | 19.5 | 6.6 | 817 | 187 | 3 | 94 | 493 | 16 | 493 | 16 |
|  | 17 | 13.7 | 12.1 | 4.8 | 466.4 | 24.1 | -0.7 | 24.8 | 19.2 | 7.3 | 19.2 | 7.3 | 918 | 216 | 3 | 98 | 569 | 17 | 569 | 17 |
|  | 18 | 14.8 | 11.8 | 4.9 | 442.1 | 24.9 | 0.9 | 24 | 19.9 | 8.7 | 19.9 | 8.7 | 959 | 217 | 5 | 99 | 593 | 20 | 593 | 20 |
|  | 19 | 19.7 | 12.5 | 4.8 | 449.7 | 30.4 | 4.7 | 25.7 | 24.4 | 13.4 | 24.5 | 13.4 | 781 | 174 | 6 | 93 | 468 | 21 | 370 | 21 |
|  | 20 | 19.4 | 12 | 4.8 | 437.9 | 29.8 | 5.2 | 24.6 | 24.1 | 13.2 | 24.1 | 13.2 | 846 | 166 | 10 | 85 | 477 | 35 | 369 | 35 |
|  | 21 | 16.3 | 12.3 | 4.6 | 485.4 | 27.1 | 0.9 | 26.2 | 21.7 | 9.5 | 21.7 | 9.5 | 1011 | 236 | 7 | 99 | 633 | 21 | 633 | 21 |
|  | 22 | 14.5 | 11.6 | 4.7 | 460 | 24.6 | 0.1 | 24.5 | 19.7 | 8 | 19.7 | 8 | 912 | 222 | 7 | 94 | 559 | 29 | 559 | 29 |
|  | 23 | 14.9 | 8.8 | 3.1 | 640.5 | 28.7 | 1 | 27.7 | 22.4 | 5.9 | 22.4 | 5.9 | 985 | 197 | 12 | 79 | 536 | 42 | 536 | 42 |
|  | 24 | 15.2 | 7.2 | 2.6 | 700.4 | 29.5 | 2.2 | 27.3 | 23.9 | 5.7 | 23.9 | 5.7 | 1075 | 170 | 20 | 64 | 493 | 64 | 493 | 64 |
|  | 25 | 17.3 | 7.4 | 2.6 | 706.6 | 31.8 | 4.2 | 27.6 | 24.3 | 7.7 | 25.9 | 7.7 | 1050 | 173 | 20 | 63 | 480 | 65 | 473 | 65 |
|  | 26 | 15.7 | 11.2 | 4.8 | 429.7 | 25.4 | 2.5 | 22.9 | 20.6 | 9.7 | 20.6 | 9.7 | 1025 | 212 | 13 | 83 | 566 | 52 | 566 | 52 |
|  | 27 | 16.4 | 11.3 | 4.8 | 435.5 | 25.9 | 2.7 | 23.2 | 21.3 | 10.3 | 21.3 | 10.3 | 1007 | 195 | 15 | 76 | 525 | 61 | 525 | 61 |
|  | 28 | 14.2 | 11.1 | 4.9 | 415.6 | 23.4 | 0.8 | 22.6 | 18.9 | 8.4 | 18.9 | 8.4 | 1029 | 218 | 13 | 87 | 583 | 45 | 583 | 45 |
|  | 29 | 20.9 | 11.2 | 4.9 | 412.3 | 30.5 | 7.8 | 22.7 | 25.3 | 16.8 | 25.3 | 15 | 922 | 181 | 11 | 81 | 516 | 41 | 516 | 41 |
|  | 30 | 24.2 | 11.2 | 4.9 | 406.1 | 34 | 11.3 | 22.7 | 28.4 | 18.3 | 28.4 | 18.3 | 873 | 153 | 11 | 71 | 443 | 47 | 395 | 47 |
|  | 31 | 19.5 | 10.9 | 4.8 | 413.6 | 29.1 | 6.8 | 22.3 | 23.9 | 13.7 | 23.9 | 13.7 | 956 | 197 | 8 | 85 | 556 | 38 | 556 | 38 |
|  | 32 | 19.2 | 10.2 | 4.8 | 395.5 | 28.4 | 7.3 | 21.1 | 23.3 | 13.6 | 23.4 | 13.6 | 941 | 187 | 10 | 76 | 504 | 45 | 432 | 45 |
|  | 33 | 16.4 | 10.6 | 4.5 | 446.4 | 25.9 | 2.7 | 23.2 | 21.2 | 10 | 21.2 | 10 | 987 | 202 | 13 | 84 | 567 | 45 | 567 | 45 |
|  | 34 | 17.6 | 10.8 | 4.6 | 448.2 | 27.4 | 4.1 | 23.3 | 22.4 | 11.1 | 22.4 | 11.1 | 939 | 189 | 12 | 81 | 531 | 45 | 531 | 45 |
|  | 35 | 17.6 | 10.7 | 4.7 | 402.7 | 27.1 | 4.8 | 22.3 | 22.1 | 12 | 22.1 | 12 | 1015 | 208 | 13 | 84 | 581 | 44 | 581 | 44 |
|  | 36 | 18.1 | 10.2 | 4.6 | 425.1 | 27.5 | 5.6 | 21.9 | 22.6 | 12 | 22.6 | 12 | 937 | 196 | 10 | 80 | 521 | 43 | 447 | 43 |
|  | 37 | 18.1 | 8.7 | 4.1 | 447.7 | 27.3 | 6.5 | 20.8 | 23 | 11.7 | 23 | 11.7 | 1045 | 207 | 12 | 76 | 554 | 45 | 554 | 45 |
|  | 38 | 16.7 | 9.4 | 4.3 | 454.6 | 25.9 | 4.2 | 21.7 | 21.7 | 10.2 | 21.7 | 10.2 | 1030 | 203 | 13 | 78 | 554 | 47 | 554 | 47 |
|  | 39 | 11.8 | 11.2 | 4.4 | 507.3 | 22.8 | -2.2 | 25 | 17.9 | 6 | 17.9 | 4.9 | 935 | 205 | 7 | 84 | 532 | 29 | 532 | 34 |
|  | 40 | 14.1 | 11.2 | 4.5 | 487.6 | 24.4 | 0 | 24.4 | 19.8 | 8.4 | 19.8 | 7.4 | 1151 | 245 | 9 | 84 | 654 | 36 | 654 | 46 |
