# Supplementary material for: Climatic Factors Drive Population Divergence and Demography: Insights Based on the Phylogeography of a Riparian Plant Species Endemic to the Hengduan Mountains and Adjacent Regions
Source: PLoS One. 2015 Dec 21;10(12):e0145014. doi: 10.1371/journal.pone.0145014 (PMC4687034; doi:10.1371/journal.pone.0145014)
Supplement: S3 Table — (DOCX) [file pone.0145014.s005.docx]

| Code | Regions | Drainages | Longitude (°E) | Latitude (°N) |
| --- | --- | --- | --- | --- |
| 1 | EH | Salween | 98.46807096 | 28.47294234 |
| 2 | HDM-YGP | Dadu River | 102.063829 | 31.476277 |
| 3 | HDM-YGP | Dadu River | 102.206504 | 31.905813 |
| 4 | HDM-YGP | Nanpan River | 102.162531 | 24.671651 |
| 5 | HDM-YGP | Red River | 101.6345556 | 24.6843518 |
| 6 | HDM-YGP | Nanpan River | 102.202461 | 24.079172 |
| 7 | HDM-YGP | Red River | 99.990101 | 26.252028 |
| 8 | HDM-YGP | Nanpan River | 102.457663 | 24.993695 |
| 9 | HDM-YGP | Jinsha River | 100.049909 | 27.186144 |
| 10 | HDM-YGP | Jinsha River | 100.394172 | 27.145776 |
| 11 | HDM-YGP | Jinsha River | 101.90208 | 25.155452 |
| 12 | HDM-YGP | Jinsha River | 102.87804 | 25.293157 |
| 13 | HDM-YGP | Jinsha River | 102.4914739 | 25.23495109 |
| 14 | HDM-YGP | Jinsha River | 102.053494 | 25.652387 |
| 15 | HDM-YGP | Jinsha River | 101.241556 | 25.5043339 |
| 17 | HDM-YGP | Mekong | 99.047226 | 27.69899 |
| 18 | HDM-YGP | Jinsha River | 102.777615 | 25.340171 |
| 19 | HDM-YGP | Jinsha River | 100.429592 | 26.79197718 |
| 20 | HDM-YGP | Jinsha River | 101.5005833 | 25.83083333 |
| 21 | HDM-YGP | Jinsha River | 101.0966111 | 25.86131944 |
| 22 | HDM-YGP | Jinsha River | 102.3739389 | 26.34055556 |
| 23 | HDM-YGP | Jinsha River | 100.4115278 | 25.94593611 |
| 24 | HDM-YGP | Jinsha River | 100.3894611 | 25.95888889 |
| 25 | HDM-YGP | Jinsha River | 100.7098056 | 26.81352778 |
| 26 | HDM-YGP | Red River | 100.0313111 | 26.07971667 |
| 27 | HDM-YGP | Jinsha River | 100.1947444 | 26.109475 |
| 28 | HDM-YGP | Jinsha River | 100.4132028 | 26.081025 |
| 29 | HDM-YGP | Red River | 100.1928306 | 25.91223056 |
| 30 | HDM-YGP | Nanpan River | 102.8174944 | 24.37286944 |
| 31 | HDM-YGP | Nanpan River | 102.8614083 | 24.49177222 |
| 32 | HDM-YGP | Nanpan River | 102.8626389 | 24.49941667 |
| 33 | HDM-YGP | Nanpan River | 102.7276667 | 24.02236111 |
| 34 | HDM-YGP | Nanpan River | 102.3910833 | 24.31661111 |
| 35 | HDM-YGP | Red River | 102.2497222 | 23.53611111 |
| 36 | HDM-YGP | Nanpan River | 103.6927778 | 23.7635 |
| 37 | HDM-YGP | Jinsha River | 101.6154722 | 26.02938889 |
| 38 | HDM-YGP | Jinsha River | 102.554575 | 25.57687222 |
| 42 | HDM-YGP | Ya-lung River | 100.650617 | 28.278134 |
| 43 | HDM-YGP | Ya-lung River | 100.843228 | 28.144714 |
| 44 | HDM-YGP | Ya-lung River | 101.125057 | 27.892828 |
| 45 | HDM-YGP | Ya-lung River | 101.2794185 | 27.9286069 |
| 46 | HDM-YGP | Ya-lung River | 100.8591856 | 28.15833616 |
| 47 | HDM-YGP | Ya-lung River | 102.07706 | 26.81287 |
| 16 | QLM | Min River | 103.590246 | 31.47677 |
| 39 | QLM | Jialing River | 104.7507389 | 32.856 |
| 40 | QLM | Jialing River | 104.2261417 | 33.29275 |
| 41 | QLM | Jialing River | 105.1108194 | 33.69644722 |
| 48 | QLM | Jialing River | 106.366278 | 33.250676 |
| 49 | QLM | Jialing River | 105.83658 | 33.332686 |
| 50 | QLM | Jialing River | 104.812418 | 33.427764 |
| 51 | QLM | Jialing River | 105.50905 | 33.422959 |
| 52 | QLM | Jialing River | 104.037409 | 33.898445 |
| 53 | QLM | Jialing River | 104.8379639 | 32.78464167 |
| 54 | QLM | Jialing River | 104.7572611 | 32.81095278 |
