# Supplementary material for: Climatic Factors Drive Population Divergence and Demography: Insights Based on the Phylogeography of a Riparian Plant Species Endemic to the Hengduan Mountains and Adjacent Regions
Source: PLoS One. 2015 Dec 21;10(12):e0145014. doi: 10.1371/journal.pone.0145014 (PMC4687034; doi:10.1371/journal.pone.0145014)
Supplement: S4 Table — (DOCX) [file pone.0145014.s006.docx]

| Haplotypes | *trn*T^(GGU)^-*psb*D | |  | *pet*L-*psb*E |  | *trn*D - *trn*T^(GGU)^ | | |
| --- | --- | --- | --- | --- | --- | --- | --- | --- |
|  | 143 | 250 |  | 64-67 |  | 30 | 122-126 | 673 |
| H1 | A | A |  | 1^a^ |  | C | 1^c^ | C |
| H2 | A | A |  | 1^a^ |  | A | 1^c^ | G |
| H3 | C | A |  | 1^a^ |  | A | 1^c^ | G |
| H4 | A | A |  | 1^a^ |  | A | 1^c^ | C |
| H5 | A | A |  | 1^a^ |  | C | 0 | C |
| H6 | A | T |  | 1^b^ |  | C | 1^c^ | C |

^a^ GGAA, ^b^TTCC, ^c^ATATT
