# Supplementary material for: Climatic Factors Drive Population Divergence and Demography: Insights Based on the Phylogeography of a Riparian Plant Species Endemic to the Hengduan Mountains and Adjacent Regions
Source: PLoS One. 2015 Dec 21;10(12):e0145014. doi: 10.1371/journal.pone.0145014 (PMC4687034; doi:10.1371/journal.pone.0145014)
Supplement: S5 Table — (DOCX) [file pone.0145014.s007.docx]

| Haplotypes | 129 | 132 | 167 | 189 | 201 | 259 | 271 | 337 | 340 | 388 | 406 | 408 | 411 | 422 | 424 | 434 | 441 | 452 |
| --- | --- | --- | --- | --- | --- | --- | --- | --- | --- | --- | --- | --- | --- | --- | --- | --- | --- | --- |
| Hap1 | G | G | A | A | T | C | G | G | A | C | A | C | T | C | C | G | A | A |
| Hap2 | G | G | A | A | T | T | G | G | A | T | T | C | T | C | C | G | A | T |
| Hap3 | G | G | A | A | T | C | G | G | A | C | T | C | T | C | T | A | A | A |
| Hap4 | G | G | A | A | T | C | G | G | A | T | A | C | T | C | C | G | A | T |
| Hap5 | G | A | A | A | T | T | G | G | A | C | T | C | T | C | T | A | A | A |
| Hap6 | G | G | A | A | A | T | G | G | A | C | T | C | T | C | T | A | A | A |
| Hap7 | G | G | A | A | T | T | G | G | A | C | T | T | T | C | T | A | A | A |
| Hap8 | G | G | G | A | T | C | G | G | A | C | T | C | T | C | T | A | A | A |
| Hap9 | G | G | A | A | T | T | G | G | A | C | T | C | T | T | T | A | A | A |
| Hap10 | G | G | A | A | T | C | G | G | A | C | T | C | T | T | T | A | A | A |
| Hap11 | G | G | A | A | T | C | G | G | A | C | T | C | T | C | T | A | G | A |
| Hap12 | G | G | A | G | T | C | C | G | C | C | T | C | T | C | T | A | A | A |
| Hap13 | G | G | A | A | T | T | G | A | A | C | T | C | C | C | T | G | A | A |
| Hap14 | G | G | A | A | T | C | G | G | A | C | A | C | T | C | C | G | A | T |
| Hap15 | T | G | A | A | T | C | G | G | A | C | T | C | T | T | T | A | A | A |
